# 5^e^ journée des travaux scientifiques des Soignants de Guyane. Nos Soignants ont du talent ! 19 & 20 Mai 2022, Cayenne, Guyane

**DOI:** 10.48327/mtsi.v2i2.2022.248

**Published:** 2022-06-30

**Authors:** Aude LUCARELLI, Louise HUREAU-MUTRICY, Blandine SOLIGNAT, Morgane BOURNE-WATRIN, Théo BLAISE, Lindsay OSEI, Pauline COUSIN, Mathilde BOUTROU, Justin DESTOOP, Alexis FREMERY, Rémi MUTRICY, Timothée BONIFAY, Loïc EPELBOIN

**Affiliations:** 1 COREVIH Guyane & CIC INSERM 1424, Centre hospitalier de Cayenne Andrée Rosemon, Guyane, France; 2 Institut de formation en soins infirmiers de Cayenne (IFSI), Guyane, France; 3 Service de dermatologie, Centre hospitalier de Cayenne, Guyane, France; 4 Centre d'investigation clinique Antilles Guyane (CIC INSERM 1424), Centre hospitalier de Cayenne, Guyane, France; 5 Service de pédiatrie, Centre hospitalier de Cayenne, Guyane, France; 6 Centre délocalisé de prévention et de soins de Grand Santi, Centre hospitalier de Cayenne, Guyane, France; 7 Unité des maladies infectieuses et tropicales, Centre hospitalier de Cayenne, Guyane, France

**Keywords:** Soignants, Personnels de santé, Santé publique, Médecine tropicale, Epidémiologie, Santé sexuelle, Périnatalité, Zoonoses, Santé globale, Addictions, Violences, Interculturalité, Covid-19, Hésitation vaccinale, Phytothérapie, Guyane, Caregivers, Health workers, Public health, Tropical medicine, Epidemiology, Sexual health, Perinatal care, Zoonoses, Global health, Addictions, Violence, Interculturality, Covid-19, Vaccine hesitation, Herbal medicine, French Guiana

Ces 19 et 20 mai 2022 se sont tenues à Cayenne, en Guyane, les 5^e^ journées des travaux scientifiques des soignants de Guyane « Nos soignants ont du talent ». Ces dernières ont remplacé depuis 2021 les Journées des travaux scientifiques des jeunes médecins de Guyane « Nos internes ont du talent », en s'ouvrant largement à d'autres corps de métier de la santé. Ainsi, lors de cette nouvelle session, 10 infirmières, 4 sages-femmes, 1 pharmacienne, 1 technicienne de laboratoire et 22 médecins ont contribué aux communications orales et affichées de ces journées. Les sujets abordés ont touché des domaines extrêmement variés, avec des travaux concernant les zoonoses (rage, fièvre Q), les interactions entre l'homme avec la faune sauvage (envenimation par la faune sauvage, en particulier ophidienne), la santé publique en zone isolée (Covid, VIH, paludisme), l’épidémiologie tropicale (hémopathies liées à l'HTLV, histoplasmose pulmonaire, toxoplasmose, tuberculose, fièvre jaune), la dermatologie tropicale (prurigo du VIH, dermo-hypodermites, dermatologie en zone isolée, leishmaniose cutanée), la périnatalité (exposition au plomb chez les femmes enceintes, causes de mort fœtale in utero, dépistage du cancer du col de l'utérus), la santé notamment sexuelle, mais aussi globale et addictive de différentes populations (orpailleurs clandestins, populations incarcérées, femmes migrantes, agriculteurs), les pathologies cardiovasculaires et les hémoglobinopathies (diabète, AVC, drépanocytose), la prise en charge des violences (épidémiologie des plaies par arme à feu, violences sexuelles et conjugales), la mise en place de projets en santé communautaire dans les quartiers défavorisés (Covid, eau-hygiène-assainissement), les soins et patients (interculturalité, barrière de la langue, EVASAN). Ces journées se sont tenues pour la deuxième fois en format mixte présentiel et/ou distanciel, avec environ 200 participants chaque jour qui ont assisté aux présentations et contribué aux débats. Enfin, le retour sur les années précédentes a montré que les travaux présentés lors de ces journées de Guyane sont loin d’être limités en termes d'intérêt scientifique à la sphère locale « guyano-guyanaise ». En effet, un pourcentage non négligeable des présentations réalisées ces dernières années a fait l'objet de publications dans des journaux internationaux principalement anglophones – 16/19 (84%) en 2017, 9/28 (32%) en 2018, 8/25 (32%) en 2019 et 10/25 (40%) en 2021. Tous les organisateurs de ces journées originales espèrent que l'implication et l'engouement des soignants pour la recherche scientifique se poursuivent, et connaissent une importance grandissante lors des sessions à venir.

**Figure 1:**
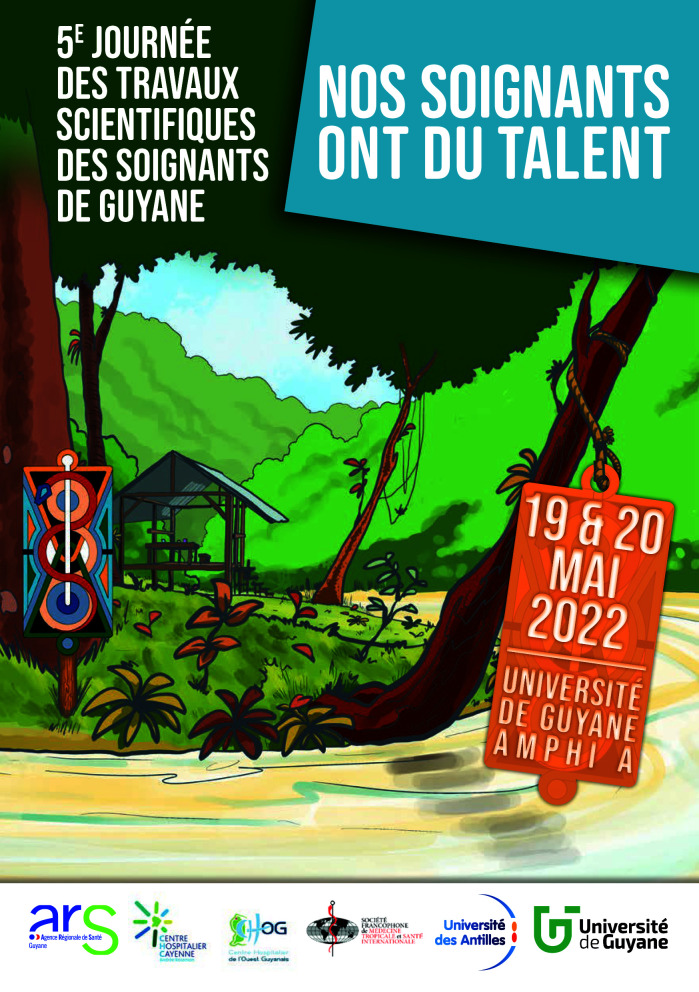
Affiche de la 5^e^ journée des travaux scientifiques des soignants de Guyane. Nos soignants ont du talent ! 19 & 20 mai 2022, Cayenne, Guyane Poster of the 5^th^ day dedicated to the scientific works of caregivers in French Guiana. Our caregivers have talent! May 19 & 20, 2022, Cayenne, French Guiana

## Remerciements

Les organisateurs tiennent à remercier Bénédicte Sauvage, directrice de Bcom, pour toute l'organisation du congrès, sans laquelle rien n'aurait été possible, Aéroprod pour la visioconférence en direct, Marie Latour et ses collaborateurs pour la mise en ligne chaque année des communications des journées, l'Agence régionale de santé Guyane pour le soutien inconditionnel jamais démenti et le financement de ces journées, le Groupement hospitalier de territoire de Guyane ainsi que l'université de Guyane pour leur soutien toujours renouvelé et enfin l'association Carbu pour l'accompagnement administratif !

