# Supplementary material for: 5th day dedicated to the scientific works of Caregivers in French Guiana. Our CAregivers have talent! May 19 & 20, 2022, Cayenne, French Guiana
Source: Med Trop Sante Int. 2022 Jun 30;2(2):mtsi.v2i2.2022.248. [Article in French] doi: 10.48327/mtsi.v2i2.2022.248 (PMC9326777; doi:10.48327/mtsi.v2i2.2022.248)
Supplement: Related texts

# Research article: La leucémie induite par le virus HTLV-1 en Guyane : données de vie réelle de 2009 à 2019

**Published:** 2022-06-30

**Authors:** Karim ABDELMOUMEN, Kinan DRAK ALSIBAI, Mathieu NACHER, Jean-Michel CAUVIN, N'detodji-Bill WANKPO, Pauline NAUDION, Caroline MISSLIN-TRITSCH, Félix DJOSSOU, Antoine GESSAIN, Lionel GALICIER, Florin SANTA, Olivier HERMINE, Jean-Pierre DROZ, Pierre COUPPIÉ, Loïc EPELBOIN

**Affiliations:** 1 Département de médecine interne, Université de La Réunion, La Réunion; 2 Département d'anatomo-pathologie, Centre hospitalier de Cayenne, Guyane, France; 3 CIC INSERM 1424, Centre hospitalier de Cayenne, Guyane, France; 4 Département d'information médicale, Centre hospitalier de Cayenne; 5 Département d'oncologie, Centre hospitalier de l'Ouest guyanais, Saint-Laurent-du-Maroni, Guyane; 6 Département des maladies infectieuses, Centre hospitalier de l'Ouest guyanais; 7 Département des maladies infectieuses, Centre hospitalier de Cayenne; 8 Épidémiologie et physiopathologie des virus oncogènes, Institut Pasteur, Paris; 9 Département d'hématologie, Hôpital Saint-Louis, Paris; 10 Département de cancérologie, Centre hospitalier de Cayenne; 11 Département d'hématologie, Hôpital Necker, Paris; 12 Professeur émérite, Département d'oncologie, Centre hospitalier de Cayenne; 13 Département de dermatologie, Centre hospitalier de Cayenne

## Introduction

La leucémie à cellules T induite par le virus HTLV-1 (ATL, Adult T-cell leukemia/lymphoma) est l'un des cancers les plus agressifs au monde. L'ATL survient chez 5 % des 10 millions de personnes vivant avec HTLV-1 dans le monde. Département français d'outremer situé dans la région amazonienne, la Guyane est l'une des principales zones d'endémie virale à HTLV-1. Les objectifs de l’étude étaient de décrire les caractéristiques démographiques, cliniques et pronostiques de la population.

## Matériel et méthodes

Nous avons collecté les données de tous les patients pour lesquels un diagnostic d'ATL a été posé entre 2009 et 2019. Les patients ont été répartis selon la classification de Shimoyama. Les variables continues ont été comparées selon un test de Mann Whitney. Les courbes de survie ont été évaluées par la méthode de Kaplan-Meier.

L’évaluation des facteurs pronostiques était basée sur un modèle de Cox.

## Résultats

Sur les 10 ans de la période d’étude, 41 patients ont été identifiés, avec un âge médian de 54 ans au diagnostic, parmi lesquels 56 % étaient des femmes. Seize (39%) patients étaient Noirs Marrons, une ethnie descendant des personnes esclavisées au sein de l'ancienne Guyane néerlandaise (Surinam). Parmi la population d’étude, 23 patients (56%) avaient une forme aiguë, 14 (34%) une forme lymphomateuse, une avait une forme chronique et une avait une forme tumorale cutanée. L'hypercalcémie était associée au sous-type aigu (69,5 % dans le groupe aigu contre 42,8 % dans le groupe lymphome; p = 0,015). Les modalités de traitement de première intention reposaient soit sur la polychimiothérapie (cyclophosphamide, doxorubicine, vincristine, prednisone), soit sur la zidovudine associée à l'interféron alpha pégylé. La survie à 4 ans était de 11,4 % pour l'ensemble de la population avec respectivement 0 % et 11 % pour les groupes lymphome et aigu. La durée médiane de survie était de 104 jours pour le groupe aigu et de 120 jours pour le groupe lymphome (p = 0,37). Les causes de décès étaient les infections (29%) et la progression de la maladie (25%). Le sexe masculin, la présence de signes « B » et l'hypercalcémie étaient associés à un mauvais pronostic avec un risque relatif (RR) de 1,4 (p = 0,479), 1,3 (p = 0,201) et 1,6 (p = 0,383) respectivement. À l'inverse, le déparasitage apparaît comme un bon facteur pronostique avec un RR de 0,680 (p = 0,394).

## Conclusion

Cette étude a fourni des données en vie réelle de patients atteints d'ATL en Guyane, un territoire à revenu élevé dans une région à revenu intermédiaire. Les patients, principalement des Noirs Marrons, se sont présentés avec un âge plus jeune au diagnostic et le pronostic était plus sombre que ce qui était attendu, par rapport aux patients japonais.

# Research article: État des lieux de la santé sexuelle des femmes migrantes consultant dans les Centres de prévention santé de la Croix-Rouge française en Guyane en 2021

**Published:** 2022-06-30

**Authors:** Leslie ALCOUFFE, Adriana GONZALEZ, Muriel VOLPELLIER, Luana BITAN, Biancaelena PANFILI, Guerline JEAN, Annette ZEPHIRIN, Pierre Marie CRETON, Florence HUBER, Nicolas VIGNIER

**Affiliations:** 1 Réseau Kikiwi, Guyane, France; 2 Croix-Rouge française de Guyane; 3 Université des Antilles; 4 COREVIH Guyane & CIC INSERM 1424, Centre hospitalier de Cayenne

## Introduction

La Guyane fait face à des enjeux démographiques et migratoires importants pour lesquels la santé de la femme devrait être prise en compte. L'objectif principal de cette étude est de décrire les situations de vulnérabilité sexuelle parmi les femmes migrantes en Guyane.

## Méthode

Cette enquête est une enquête transversale de méthodologie mixte, comportant :

- un volet épidémiologique descriptif transversal, par questionnaires administrés en français, créole haïtien, espagnol et portugais, auprès d'un échantillon aléatoire de femmes de plus de 18 ans nées à l’étranger consultant dans les Centres de prévention santé (CPS) de la Croix-Rouge à Saint-Laurent-du-Maroni et Cayenne d'avril à août 2021;
- un volet exploratoire qualitatif, à travers des entretiens semi-directifs menés auprès des soignants intervenants dans les mêmes centres durant la même période.

## Résultats

Un total de 382 femmes a été inclus, âgées en médiane de 31 années, originaires principalement d'Haïti (80,4%), du Suriname (8,9%) et de République dominicaine (5,7%), et 12 entretiens ont été menés auprès des équipes de soins. Les soignants ont rapporté une exposition élevée à la violence sexuelle chez les femmes consultant au sein des CPS, ainsi qu'un recours au sexe transactionnel important. Parmi les 382 femmes interrogées, 19,9 % déclarent avoir été violées au cours de leur vie, 27,7 % avoir déjà accepté un rapport sexuel en échange d'argent et 27,6 % avoir déjà subi des violences physiques. Les équipes de soins ont rapporté un état de santé mental dégradé chez les consultantes; les troubles anxieux et dépressifs ont été fréquemment retrouvés dans le volet épidémiologique.

Ces éléments sont à mettre en regard de la précarité dans laquelle les femmes évoluent : 76,7 % des enquêtées décrivant une situation financière difficile, et 55,5 % exposées à une faim sévère. Concernant le réseau de soutien, 72,5 % ont déclaré n'avoir personne pour les héberger en cas de besoin. Cette précarité est aussi menstruelle : 73,7 % des femmes ont déclaré avoir manqué de protections hygiéniques au cours des dernières semaines.

## Conclusion

La vulnérabilité sexuelle des femmes migrantes en Guyane est préoccupante et semble liée à la précarité dans laquelle celles-ci évoluent. Les mesures d'urgence (aide alimentaire, gratuité des protections hygiéniques, etc.) et de moyen terme (interventions sociales et communautaires de réduction des risques sexuels, prise en charge psychologique) méritent d’être renforcées.

# Research article: Le jeûne du mois de Ramadan chez le patient diabétique en Guyane d'avril 2019 à juin 2019 : étude descriptive sur une patientèle de ville

**Published:** 2022-06-30

**Authors:** Awatef ARGOUBI, Nadia SABBAH

**Affiliations:** 1 Ville de Cayenne, Guyane, France; 2 Service d'endocrinologie et de maladie métabolique, Centre hospitalier de Cayenne Andrée Rosemon, Guyane, France; 3 Centre d'investigation clinique, Centre hospitalier de Cayenne Andrée Rosemon, Université Antilles Guyane (CIC INSERM 1424), Cayenne, Guyane, France

## Contexte & Introduction

Dans le cadre du DU de diabétologie et face à une situation rencontrée dans le cadre de mon exercice professionnel, j'ai été amené à me poser certaines questions relatives à l'impact des croyances sur l'observance et l'impact réel en Guyane avec l'arrivée de nouvelles populations sur le territoire.

## Méthodologie

Questionnaires auprès d'un petit échantillon populationnel.

## Principaux résultats

Tous les patients sont conscients qu'il existe un risque à jeûner. Ils évoquent difficilement avec les professionnels de santé ce moment particulier dans leur vie religieuse; ils en parlent par contre facilement à leur entourage proche (famille, amis), sur les réseaux sociaux ainsi qu’à leur médecin traitant. Personne n'a évoqué le risque de déshydratation en Guyane qui aurait pu résulter des températures plutôt élevées. Ceux qui ont vécu en métropole au contraire se réjouissent de jeûner en Guyane, où la durée de jeûne est plus courte. De ce fait, ils se sentent moins en difficulté par rapport aux années précédentes en France. Les professionnels de santé ont un rôle important à jouer pour sensibiliser les patients diabétiques aux risques encourus et leur offrir des lignes directives afin de minimiser les effets néfastes qui pourraient résulter de leur jeûne. Les traitements et les conseils sont à adapter à chaque personne. Les patients qui en ont discuté avec leur médecin traitant ou infirmière avaient pour point commun que le praticien était musulman. En Guyane, où les professionnels de santé ne sont peut-être pas familiers avec le jeûne, la recommandation de ne pas jeûner n'est pas suivie. Il y a peut-être un manque de communication.

## Discussion & Conclusion

La prise en charge de ces patients doit être personnalisée (au cas par cas) avec :

1 Bilan de la situation du diabétique et évaluation des risques de complications (antécédents).
2 Éducation : proposition de séances d’éducation thérapeutique pré-Ramadan à tous les patients, qu'ils pratiquent le jeûne ou non.
3 Augmentation de la surveillance de la glycémie capillaire, surtout chez les patients traités par insuline.
4 Conseils nutritionnels sur la composition du repas du soir. Introduction de plus de légumes et de fruits (fibres). Le repas du matin doit être retardé le plus possible avec un apport suffisant.
5 Conseils sur l'activité physique : adapter l'activité au jeûne et renforcer la surveillance glycémique. À noter que les prières du soir doivent être considérées comme une activité physique.
6 Éducation sur les conditions de rupture de jeûne de façon systématique et immédiate :
  - en cas d'hypoglycémie (glycémie < 0,60 g/l : arrêt immédiat et urgent et resucrage)
  - si glycémie < 0,70 g/l : rupture du jeûne
  - si hyperglycémie
  - s'il ne se sent pas bien
7 Adaptation des traitements avec le spécialiste ou le médecin traitant :
  - Antidiabétiques oraux
  - Insuline
  - Au cas par cas selon les dernières recommandations

# Research article: Proportion des lésions de haut grade du col de l'utérus chez les femmes de moins de 25 ans en Guyane

**Published:** 2022-06-30

**Authors:** Pascaline AVILON-HECKMANN, Kinan DRAK ALSIBAI, Fabrice QUET, Adrien EHRHARDT, Nadia THOMAS

**Affiliations:** 1 Service de gynécologie-obstétrique, Centre hospitalier de Cayenne Andrée Rosemon, Guyane, France; 2 Service d'anatomie et cytologie pathologiques, Centre hospitalier de Cayenne, Guyane, France; 3 INSERM CIC, Centre hospitalier de Cayenne, Guyane, France; 4 Centres délocalisés de prévention et de soins (CDPS), Centre hospitalier de Cayenne, Guyane, France

## Contexte & Introduction

En Guyane française, le cancer du col de l'utérus est le deuxième cancer le plus fréquent chez la femme (après le cancer du sein); son incidence est 3 fois plus élevée qu'en France hexagonale. Le diagnostic se fait en moyenne chez des femmes plus jeunes (47 *vs* 51 ans) et à un stade plus avancé. Devant ce constat, un dépistage anticipé par frottis cervicoutérin (FCU) est suggéré à partir de 20 ans au lieu de 25 ans comme sur le plan national. L'objectif de cette étude est d’établir la proportion de lésions cytologiques de haut grade du col de l'utérus chez les femmes de moins de 25 ans ayant bénéficié d'un FCU en Guyane sur une période de 10 ans.

## Méthodologie

Nous avons mené une étude descriptive rétrospective transversale. La population d’étude regroupait toutes les patientes de moins de 25 ans ayant bénéficié d'un FCU, quel que soit le motif de consultation, entre le 1er janvier 2010 et le 31 décembre 2019 en Guyane.

## Principaux résultats

Après exclusions, 17 509 frottis ont été pris en compte pour l'analyse des lésions malpighiennes. 15 421 ne présentaient pas d'anomalie soit 89,27 %; 1 854 FCU présentaient une anomalie malpighienne soit 10,73 % dont 4,09 % ASCUS, 5,77 % LIEBG, 0,41 % ASCH et 0,47 % LIEHG. Aucun cancer n'a été retrouvé.

## Discussion & Conclusion

Il s'agit de la première étude traitant des lésions du col de l'utérus chez les moins de 25 ans en Guyane. Au vu des résultats et en termes de balance bénéfice/risque, le dépistage généralisé anticipé à partir de 20 ans ne semble pas indiqué.

# Research article: Victimes de plaies par arme à feu au Centre hospitalier de Cayenne : étude épidémiologique de 2016 à 2019

**Published:** 2022-06-30

**Authors:** Elliott BEGUINOT, Alexis FREMERY, Mathilde DOUCHET, Jean PUJO, Hatem KALLEL

**Affiliations:** 1 Service d'accueil des urgences, Centre hospitalier de Cayenne, Guyane, France; 2 Service de réanimation polyvalente, Centre hospitalier de Cayenne

## Contexte

Les plaies par arme à feu sont peu étudiées et sont responsables d'une mortalité élevée. La Guyane est le territoire français où le taux d'agression armée est le plus élevé. Elle présente également une situation géographique et sociodémographique singulière rendant le système de soin particulier. L’étude épidémiologique des traumatismes balistiques permettrait d'améliorer la prise en charge de ces patients. L'objectif de ce travail est d’établir l’épidémiologie des victimes par arme à feu prises en charge à Cayenne afin de définir les caractéristiques sociodémographiques de celles-ci et les facteurs associés au décès.

## Méthode

De janvier 2016 à décembre 2019, nous avons réalisé une étude rétrospective au Centre hospitalier de Cayenne (CHC), incluant tous patients admis pour le motif de plaies par arme à feu au service d'accueil des urgences, au SAMU et à l'Unité médico-judiciaire (UMJ).

## Résultats

Notre étude a inclus 340 patients, dont 71 victimes décédées. Notre cohorte était composée majoritairement d'une population d'hommes (90%), jeunes (30 ± 11 ans). Les événements se sont déroulés principalement la nuit (60%), dans un contexte d'agression (83%) et en lien avec l'utilisation d'armes d’épaule (82%). Parmi les 290 patients pris en charge au CHC, 60 % ont été hospitalisés dont 12 % en réanimation, 41 % ont nécessité un traitement chirurgical et 7 % sont décédés. La durée moyenne de séjour (DMS) globale était de 10 ± 18 jours. Nous n'avons pas retrouvé de différence significative sur les critères de gravité (p = 0,23) ou de décès (p = 0,64) entre les victimes de Cayenne et ses environs et celles des communes isolées. Seuls les délais d'admission (0h59 *vs* 8h15, p < 0,001) et la DMS (8 j *vs* 19 j, p < 0,001) étaient statistiquement augmentés pour les victimes issues des sites isolés. La mortalité globale (n = 71) était associée au sexe (p < 0,001) et au contexte de suicide (p < 0,001). Les orifices d'entrée (tête et cou, thorax; p < 0,001) ainsi que les lésions induites (neurologiques, respiratoires et vasculaires; p < 0,005) se sont révélés comme des facteurs prédictifs de mortalité.

## Conclusion

Ce travail souligne la forte incidence des traumatismes balistiques en Guyane. Celle-ci touche principalement une population jeune et masculine en lien avec la circulation d'armes d’épaule et les agressions. Malgré les difficultés géographiques liées au territoire et les déficits de plateau technique, la mortalité est comparable à d'autres études notamment en Guadeloupe, mais reste plus de deux fois supérieure à la France hexagonale. Ce travail soulève de nombreux axes d'amélioration possibles, notamment sur la prise en charge et les moyens de prévention disponibles.

# Research article: Exposition au plomb des patientes enceintes suivies en Centres délocalisés de prévention et de soins (CDPS) sur l'Oyapock et le Haut-Maroni de 2018 à 2020 : quel impact sur la grossesse ? Étude EPEOMIG

**Published:** 2022-06-30

**Authors:** Noémie BERTON, Nadia THOMAS, Aurel CARBUNAR, Alexane GUYOMARD

**Affiliations:** 1 Sages-femmes échographistes, Centre hospitalier de Kourou, Guyane, France; 2 Gynécologue-obstétricien, Centre hospitalier de Cayenne Andrée Rosemon, Guyane, France; 3 Centres délocalisés de prévention et de soins (CDPS), Centre hospitalier de Cayenne Andrée Rosemon, Guyane, France; 4 Statisticien épidémiologiste, Association IDsanté, Cayenne, Guyane, France

## Introduction

La prévalence de l'intoxication au plomb chez les femmes enceintes suivies en Centres délocalisés de prévention et de soins (CDPS) dans les communes isolées de l'intérieur dela Guyane est inconnue. Il existe peu de données sur le saturnisme pendant la grossesse en Guyane. Notamment, la prévalence de l'exposition au plomb chez les femmes enceintes est inconnue. L'un des objectifs de cette étude est d'estimer cette prévalence ainsi que d'identifier les principales conséquences obstétricales et fœtales associées à cette exposition chez les populations exposées ou non au plomb.

## Matériel et méthode

EPEOMIG est une étude rétrospective multicentrique observationnelle et descriptive réalisée à partir des données recueillies dans les dossiers médicaux. La population étudiée correspondait aux femmes majeures suivies dans les CDPS de Saint-Georges et Maripasoula ayant un début de grossesse compris entre mars 2018 et septembre 2020.

## Résultats

261 femmes ont été incluses dans cette étude. La moyenne de la plombémie était de 32,8 µg/L, quatre fois supérieure au taux de plombémie retrouvé en France métropolitaine en 2011.

L’étude a mis en évidence la grande prévalence du saturnisme dans nos populations puisqu'un tiers des patientes en sont atteintes. Elle a également permis de mettre en évidence des liens significatifs entre saturnisme et complications obstétricales. En effet, plus la plombémie était élevée, plus le risque d'issues de grossesse défavorables augmentait (p = 0,004). On retrouvait chez les patientes atteintes de saturnisme davantage de retards de croissance intra-utérins (RCIU) (p = 0,032), davantage d'enfants dont le poids de naissance était inférieur au 10^e^ percentile (p = 0,017) et plus d'antécédents de perte fœtale (p = 0,017).

## Discussion

Ces résultats mettent en évidence un véritable problème de santé publique dans les communes de l'intérieur de la Guyane. Les précédentes études qui avaient été réalisées sur le littoral n'avaient pas permis de conclure à des liens significatifs. D'autres études de plus grande envergure sont nécessaires afin d’évaluer les effets de l'imprégnation au plomb, particulièrement chez les patientes amérindiennes qui sont les plus exposées.

# Research article: Leishmaniose cutanée et co-infections parasitaires dans une commune amérindienne de Guyane

**Published:** 2022-06-30

**Authors:** Bastien BIGEARD, Denis BLANCHET, Magalie PIERRE-DEMAR, Thomas MALMONTET, Pierre COUPPIÉ, Romain BLAIZOT

**Affiliations:** 1 Service des maladies infectieuses et tropicales, Centre hospitalier universitaire de Martinique, route de Chateauboeuf, 97200 Fort-de-France, France; 2 Laboratoire hospitalo-universitaire de parasitologie-mycologie, Centre hospitalier de Cayenne, Guyane, France; 3 Service de dermatologie, Centre hospitalier de Cayenne, Guyane, France

## Contexte & Introduction

Plusieurs études ont montré l'existence d'interactions entre certaines maladies parasitaires, semblant être médiées par l'immunité. Les protozooses comme la leishmaniose, le paludisme, la toxoplasmose et la trypanosomiase américaine mais également les helminthiases ont une incidence élevée en Guyane. L'objectif de notre étude était de rechercher des interactions entre la survenue d'une leishmaniose cutanée ou muqueuse et les co-infections parasitaires dans une commune amérindienne du Haut-Oyapock en Guyane.

## Méthodologie

Nous avons réalisé une étude comparative, rétrospective, dans les Centres délocalisés de prévention et de soins (CDPS) de Camopi et Trois-Sauts sur le Haut-Oyapock en Guyane. Les patients étaient inclus en cas de leishmaniose cutanée ou muqueuse survenue entre le 1er janvier 2008 et le 31 décembre 2018. Nous avons recherché dans les dossiers médicaux des patients ainsi que dans les bases de données du CH de Cayenne toute infection parasitaire documentée : paludisme, toxoplasmose, trypanosomiase américaine ou parasitose digestive. Les données ont été comparées à l'incidence de chaque parasitose dans la population d’étude chez les patients n'ayant pas d'antécédent de leishmaniose.

## Principaux résultats

Soixante-quatre patients ont été inclus dont 8 ayant eu au moins un épisode de paludisme avant la leishmaniose, 2 ayant eu une toxoplasmose après la leishmaniose, et 4 ayant eu au moins un épisode de parasitose digestive, dont 2 avant la leishmaniose et 2 après. Nous avons observé une diminution significative des cas de paludisme chez les patients ayant un antécédent de leishmaniose (OR 0,12; IC 95 % [0,01-0,68], p = 0,010).

## Discussion & Conclusion

La survenue d'une leishmaniose cutanée ou muqueuse en Guyane semble associée à une diminution des cas de paludisme chez les Amérindiens du Haut-Oyapock. Cette observation pourrait être expliquée par une stimulation de l'immunité Th1 en réponse à l'infection par *Leishmania,* rendant l'environnement hostile à l'implantation du *Plasmodium.* Un suivi prolongé des cas index de leishmaniose ainsi que des études sur le plan immunologique seraient nécessaires afin d’étayer ces premiers résultats.

# Research article: Histoplasmose pulmonaire chez les personnes vivant avec le VIH en Guyane : cohorte rétrospective sur 31 ans

**Published:** 2022-06-30

**Authors:** Morgane BOURNE-WATRIN, Antoine ADENIS, Gary DOPPELT, Jeanne BIGOT, Kinan DRAK ALSIBAI, Romain BLAIZOT, Carl LAMOUREUX, Justin DESTOOP, Denis BLANCHET, Magalie DEMAR, Loïc EPELBOIN, Mathieu NACHER, Geneviève GUILLOT, Magaly ZAPPA, Félix DJOSSOU, Pierre COUPPIÉ

**Affiliations:** 1 Service de dermatologie-vénérologie, Centre hospitalier de Cayenne, Guyane, France; 2 Centre d'investigation clinique Antilles Guyane (CIC INSERM 1424), Centre hospitalier de Cayenne, Guyane, France; 3 Tropical Biome and Immunophysiopathology (TBIP), Université de Guyane, Université de Lille, CNRS, INSERM, Institut Pasteur de Lille, U1019-UMR9017-CIIL Centre d'infection et d'immunité de Lille, France; 4 Service de radiologie, Centre hospitalier de Cayenne, Guyane, France; 5 Service de parasitologie-mycologie, Centre hospitalier Saint Antoine APHP, Paris, France; 6 Laboratoire d'anatomie et cytologie pathologiques, Centre hospitalier de Cayenne, Guyane, France; 7 Laboratoire de parasitologie-mycologie, Centre hospitalier de Cayenne, Guyane, France; 8 Unité des maladies infectieuses et tropicales, Centre hospitalier de Cayenne, Guyane, France; 9 Service de Médecine B, Centre hospitalier de Cayenne, Guyane, France

## Introduction

Au cours du syndrome d'immunodéficience acquise (sida), l'histoplasmose se présente sous une forme disséminée dans 95 % des cas. La méconnaissance de son atteinte pulmonaire sur le plan radiologique chez les personnes vivant avec le virus de l'immunodéficience humaine (PVVIH) conduit à des diagnostics erronés de tuberculose ou pneumocystose. Notre objectif était de décrire l'atteinte pulmonaire de l'histoplasmose dans cette population.

## Méthodologie

Une étude rétrospective multicentrique a été menée entre janvier 1988 et octobre 2019 chez les PVVIH adultes en Guyane. L'histoplasmose pulmonaire était documentée par examen direct mycologique, culture ou histologie. Les patients présentant d'autres infections pulmonaires concomitantes étaient exclus.

## Principaux résultats

Soixante-cinq patients ont étéinclus. Lesex-ratioH/Fétaitde2,4etl’âge médian était de 39 ans. Aucun ne présentait de contrôle immuno-virologique de son infection. Le nombre médian de CD4 était de 24/mm^3^. L'histoplasmose était l’événement classant SIDA chez 88 % des patients avec 29 % d'autres événements classant SIDA concomitants. La présentation clinique retrouvait fièvre (89%), toux (59%), dyspnée (35%), expectorations (14%) et hémopty-sie (5%). Soixante-et-une radiographies et 24 scanners ont été réalisés. La présentation radiogra-phique la plus fréquente était un syndrome interstitiel (77%). Sur le scanner 92 % des patients présentaient un syndrome nodulaire, principalement sous la forme de micronodules diffus (71%) parmi lesquels 54 % de miliaires et un signe du cluster. Les autres anomalies comprenaient nodules et macronodules (70%), adénopathies thoraciques (63%), septa intralobulaires (50%), foyers alvéolaires (29%), cavernes et épanchements pleuraux (25 % chacun), opacités en verre dépoli et kystes (15 % chacun). Malgré un traitement antifongique approprié, la mortalité à un mois était de 22 %.

## Conclusion

Face à un syndrome interstitiel radiographique ou une miliaire scannographique chez des PVVIH non contrôlées, le diagnostic d'histoplasmose doit être évoqué au même titre que la tuberculose ou la pneumocystose en zone d'endémie.

# Research article: Prurigo du patient VIH et traitement antirétroviral

**Published:** 2022-06-30

**Authors:** Albane CALAS, Basma GUARMIT, Mathieu NACHER, Pierre COUPPIÉ

**Affiliations:** 1 Service de dermatologie-vénérologie, Centre hospitalier de Cayenne, Guyane, France; 2 Centre d'investigation clinique Antilles Guyane (CIC INSERM 1424), Centre hospitalier de Cayenne, Guyane, France; 3 Tropical Biome and Immunophysiopathology (TBIP), Université de Guyane, Université de Lille, CNRS, INSERM, Institut Pasteur de Lille, U1019-UMR9017-CIIL Centre d'infection et d'immunité de Lille, France

## Introduction

Le prurigo est une manifestation dermatologique chez les personnes vivant avec le virus de l'immunodéficience humaine (PVVIH). Le traitement, non standardisé, repose sur une trithérapie antirétrovirale (ARV). L'IRIS *(Immune reconstitution inflammatory syndrome)* regroupe l'ensemble des manifestations attribuées à la restauration d'une réponse immunitaire excessive secondaire au traitement ARV.

## Étude 1

### Objectif

Décrire la durée d’évolution du prurigo après introduction d'ARV chez les

PVVIH.

### Méthodologie

Étude rétrospective menée entre 1996 et 2017 chez les PVVIH suivies au Centre hospitalier de Cayenne, Guyane, France (CHC) ayant un prurigo diagnostiqué cliniquement dans les 6 mois précédant l'introduction d'un traitement ARV.

### Résultats

51 patients ont été inclus. La médiane de disparition du prurigo sous traitement était de 3,0 mois [0,5-54].

### Discussion

Le délai de disparition du prurigo sous traitement ARV est peu décrit dans la littérature. L'hétérogénéité des durées dans notre échantillon pourrait s'expliquer par certains prurigos non liés au VIH.

## Étude 2

### Objectif

Décrire le délai d'apparition du prurigo après introduction d'ARV chez les PVVIH sans prurigo initial.

### Méthodologie

Étude rétrospective menée entre 1996 et 2017 chez les PVVIH suivies au CHC ayant un prurigo apparu dans les 6 mois suivant l'introduction d'un traitement

ARV.

### Résultats

32 patients ont été inclus avec un délai d'apparition médian du prurigo de 1,4 mois, significativement associé au type de traitement ARV. La durée d’évolution médiane avant disparition était de 3 mois.

### Discussion

L'apparition de prurigo sous traitement ARV évoque un IRIS, sans pouvoir exclure une toxicité cutanée liée aux ARV. La durée d’évolution médiane du prurigo sous ARV était similaire à celle de notre première étude.

### Conclusion

Tout prurigo doit faire rechercher une infection par le VIH. Son traitement repose sur un traitement ARV avec des délais de disparition hétérogènes. L'apparition d'un prurigo chez un patient VIH sous traitement ARV évoque un phénomène IRIS.

# Research article: L'infirmière (IDE) d’éducation thérapeutique, coordinatrice de parcours des personnes vivant avec le VIH (PVVIH) sur le bassin de l'Oyapock – Projet « Oyapock Coopération Santé » (OCS)

**Published:** 2022-06-30

**Authors:** Mylène CEBE, Sophie RHODES, Jonailde COADOU, Jane MILLER, Margaux BARBIER, Bastien BIDAUD, Céline MICHAUD

**Affiliations:** 1 Centre hospitalier de Cayenne, Guyane, France; 2 Association IDsanté, Cayenne, Guyane; 3 Association DAAC Guyane, Saint-Georges-de-l'Oyapock, Guyane; 4 Association DPAC Fronteira, Oiapoque, Brésil

## Contexte & Introduction

La frontière entre la Guyane et le Brésil est marquée par le fleuve Oyapock. D'un côté se trouve la ville française de Saint-Georges et de l'autre, la ville brésilienne d'Oiapoque. L'une comme l'autre sont isolées géographiquement et disposent d'un accès limité aux soins. Le turn-over du personnel soignant y est important. Ce bassin de vie est un lieu de croi-sement de populations vulnérables. L’épidémie de VIH n'y est pas contrôlée.

## Méthodologie

Dans le cadre du projet transfrontalier OCS, une IDE est en charge du volet « Prévention combinée ». Cette professionnelle fait le lien entre tous les intervenants concernés par le parcours de soins des PVVIH en étant une interlocutrice privilégiée et ressource pour les patients. Elle a pour missions la gestion de la file active des PVVIH, la prévention et le dépistage au sein du CDPS (Centre délocalisé de prévention et de soins) et hors les murs, la création d'un réseau transfrontalier autour du patient avec un soutien à la prise en charge à Oiapoque au sein du dispositif de la « première ligne ».

## Principaux résultats

Un suivi des PVVIH a débuté en mars 2019 auprès de la première ligne d'Oiapoque (hors mineurs, femmes enceintes, stade Sida). En 3 ans, 74 PVVIH ont intégré la prise en charge dont 32 transferts depuis Saint-Georges.

L'IDE OCS poursuit le suivi de la file active au sein du CDPS de Saint-Georges (31 en février 2022), la recherche et la remise dans le soin des perdus de vue et les actions de prévention. Elle participe à l'animation du lien des intervenants franco-brésiliens.

## Discussion & Conclusion

Les zones frontalières constituent des zones clés et précarité où il faut adapter les prises en charge afin de mieux contrôler l’épidémie. L'IDE OCS est un exemple réussi et innovant de processus collaboratif opérationnel transfrontalier.

# Research article: Évaluation de l'activité de l'Unité d'hospitalisation de courte durée des Urgences du Centre hospitalier de Cayenne, Guyane, France en 2018 et 2019

**Published:** 2022-06-30

**Authors:** Aurélien DEBELLE, Jean-Michel CAUVIN, Héloïse DEBELLE, Hatem KALLEL, Rémi MUTRICY

**Affiliations:** 1 Service d'accueil des urgences, Centre hospitalier de Cayenne, Guyane, France; 2 Département universitaire de médecine générale, Université des Antilles, Pointe-à-Pitre, Guadeloupe, France; 3 Département d'information médicale, Centre hospitalier de Cayenne, Guyane, France; 4 Research Institute for Sport and Exercise Sciences, Liverpool John Moores University, Liverpool, Royaume-Uni; 5 Service de réanimation, Centre hospitalier de Cayenne, Guyane, France

## Introduction

En France, les Unités d'hospitalisation de courte durée (UHCD) sont, depuis 1991, définies comme faisant partie intégrante des services d'urgence. Depuis 2001, des recommandations de bonne pratique ont été émises par la Société française de médecine d'urgence (SFMU). Malgré une définition précise, plusieurs études ont montré une utilisation non appropriée de cette unité.

L'objectif principal de cette étude est de vérifier si l'UHCD du Centre hospitalier de Cayenne, Guyane, France est en adéquation avec les critères définis.

## Méthodologie

Nous avons réalisé une étude rétrospective et descriptive de l'intégralité des Résumés d'unité médicale (RUM) de l'UHCD entre le 1er janvier 2018 et le 31 décembre 2019.

Les variables étudiées étaient l’âge et le sexe des patients, la durée moyenne de séjour, la classification SFMU des malades admis dans le secteur, leur gravité selon le score CCMU, le mode d'entrée et de sortie du service, les diagnostics présentés à l'entrée de l'UHCD ainsi que le taux d'occupation des lits de l'UHCD.

## Résultats

Au total, nous avons analysé 2 859 RUM. L’âge moyen des patients est de 56 ans, la durée moyenne de séjour est de 63 h et les patients restés plus de 24 h représentent 62,1 % des patients. La majorité des patients relèvent des groupes 2 et 3 selon la classification SFMU et sont essentiellement représentés par le groupe 2 du score de gravité CCMU. Le taux d'occupation des lits y est supérieur à 100 % tous les mois des années d’étude.

## Conclusion

L'UHCD du CHC n'est conforme en aucun point aux règlementations précises de bonne pratique émises par la SFMU. Des mesures correctrices ciblées sont nécessaires pour une optimisation du fonctionnement de l'UHCD.

# Research article: La santé sexuelle et reproductive des femmes francophones incarcérées en 2021 au Centre pénitentiaire de Remire-Montjoly : une approche qualitative

**Published:** 2022-06-30

**Authors:** Flore DEBOSCKER, Mathieu NACHER, Antoine ADENIS, Florence HUBER, Aude LUCARELLI, Laura ASENSIO, Marie DANIEL, Vanessa SCHIEMSKY, Timothée BONIFAY

**Affiliations:** 1 Unité sanitaire en milieu pénitentiaire, Centre hospitalier de Cayenne, Guyane, France; 2 Département universitaire de médecine générale, Université des Antilles, Pointe-à-Pitre, Guadeloupe, France; 3 Centre d'investigation clinique Antilles Guyane (CIC INSERM 1424), Centre hospitalier de Cayenne, Guyane, France; 4 Croix-Rouge française de Guyane, Guyane, France; 5 Hôpital de jour, Centre hospitalier de Cayenne, Guyane, France

## Introduction

Les femmes incarcérées constituent une population vulnérable en termes de santé sexuelle et reproductive. En Guyane, la majorité des détenues sont issues d'un milieu précaire et incarcérées pour trafic de stupéfiants. Les modalités du suivi médical carcéral ne permettent pas d’évaluer l'impact de l'incarcération sur le rapport des détenues à leur santé féminine. En l'absence de données, l'objectif de l’étude était d'interroger le vécu et les pratiques liées à la santé sexuelle et reproductive des femmes détenues.

## Méthodes

Des entretiens semi-directifs ont été menés auprès de femmes majeures et francophones, incarcérées depuis au moins 4 mois au Centre pénitentiaire de Guyane. Plusieurs thématiques (menstruations, contraception, grossesse, IVG, IST, sexualité) ont été décrites par analyse interprétative phénoménologique.

## Résultats

Quatorze femmes ont été entretenues. Elles souffraient de troubles du cycle menstruel, d'un manque d'hygiène et de précarité menstruelle. Leur sexualité apparaissait désinvestie et le plaisir féminin absent. Cependant, une sexualité intra-carcérale existe (masturbation, parloir, entre codétenues), mais les rapports homosexuels étaient source de discrimination. Les pratiques de piercing et tatouage étaient répandues, avec un faible niveau de connaissance concernant les maladies infectieuses transmissibles. Le fait d’être enceinte au cours de l'incarcération était jugé négativement. Les détenues formulaient des stratégies pour ne pas s'y exposer.

## Conclusion

L'incarcération était une période de vulnérabilité au plan de la santé sexuelle et reproductive des femmes. Une sexualité active exposait à des prises de risque et des discriminations qu'il convient de prendre en compte dans le cadre d'une approche pluridisciplinaire adaptée au milieu carcéral.

# Research article: Saisonnalité des dermo-hypodermites bactériennes non nécrosantes en milieu tropical ? Étude épidémiologique en Guyane

**Published:** 2022-06-30

**Authors:** Justin DESTOOP, Pierre COUPPIÉ

**Affiliations:** 1 Service de dermatologie-vénérologie, Centre hospitalier Andrée Rosemon, Cayenne, Guyane, France

## Méthodologie

Nous avons réalisé une étude rétrospective monocentrique et observationnelle sur les patients hospitalisés pour une DHBNN en dermatologie et infectiologie à l'hôpital de Cayenne sur 11 ans (2010-2021). Les données cliniques, sociodémographiques et les résultats microbiologiques étaient recueillis. Concernant l’étude de la saisonnalité, nous avons défini 2 périodes : la saison « humide » correspondant aux 3 mois les plus pluvieux (avril-mai-juin) et la saison « sèche » aux 3 mois les plus secs (août-septembre-octobre) selon les données recueillies par Météo France sur Cayenne.

## Résultats

Trois-cent-soixante-douze patients ont été inclus. L’âge moyen était de 60,0 ± 18,5 ans et 215 (57,8%) étaient des hommes. Cent neuf (29,3%) patients étaient obèses, 94 (25,3%) étaient diabétiques, 37 (9,9%) étaient immunodéprimés. Trois cent quarante-trois (92,2%) patients avaient des DHBNN localisées aux membres inférieurs, 20 (5,4%) aux membres supérieurs. Quatre-vingt-six (23,1%) avaient des complications locales (65 phlyctènes, 18 abcès). Treize (3,8%) patients avaient des hémocultures positives (4 à *S. pyogenes,* 7 à un autre streptocoque β hémolytique (B,C,G) et 2 à *S. aureus).* Neuf (2,4%) patients avaient une culture d'abcès positive (3 à *S. pyogenes,* 3 à *S. aureus* et 3 polymicrobiennes dont 2 associations *S. aureus* et *S. pyogenes).* Quinze (4,0%) patients avaient une culture positive de phlyctènes (2 à *S. aureus,* 1 à *S. pyogenes,* 1 à V. *vulnificus*, 3 à entérobactéries et 8 polymicrobiennes). Il existait une saisonnalité en faveur de la période sèche avec 2 fois plus de patients hospitalisés (125 *vs* 71, p = 0,0004).

## Conclusion

Le profil clinique des DHBNN hospitalisées à Cayenne était similaire aux régions non tropicales à l'exception d'une prédominance masculine. Les germes impliqués étaient majoritairement des streptocoques β hémolytiques et le *Staphylococcus aureus.* Il existait une saisonnalité avec 2 fois plus de patients hospitalisés en période sèche comparativement à la période humide.

# Research article: Étude rétrospective analysant les évacuations sanitaires réalisées lors de la première vague épidémique de Covid-19 en Guyane

**Published:** 2022-06-30

**Authors:** Mathilde DOUCHET, Hatem KALLEL, Jean-Marc PUJO, Alexis FREMERY

**Affiliations:** 1 Service d'accueil des urgences, Centre hospitalier de Cayenne, Guyane, France; 2 Service de réanimation polyvalente, Centre hospitalier de Cayenne, Guyane, France

## Introduction

La première vague de la pandémie à SARS-CoV-2 a touché notre région de juin à août 2020. Compte tenu de l'isolement de notre Centre hospitalier (CH), des évacuations sanitaires (EVASAN) aériennes vers les départements d'outre-mer (DOM) les plus proches ont été réalisées pour désengorger les services de soins intensifs. Un protocole spécifique STRATEVAC a été mis en place pour faciliter ces transferts, en collaboration avec les différents SAMU, services de réanimation, les Forces armées et les ARS. Le transfert de patients à risque infectieux était une première. L'objectif de cette étude était d'analyser ces transferts et de chercher des facteurs de risque de mortalité suite au transport aérien de ces patients à haut risque.

## Méthodologie

Cette étude observationnelle rétrospective incluait tous les patients présentant une infection à Covid-19 transférés lors de la première vague de l’épidémie et évaluait les paramètres cliniques et biologiques avant et après transport. Ces données ont été comparées selon la survie à l'issue de la prise en charge.

## Résultats

Douze patients ont été transférés par avion médicalisé dans les services de réanimation des DOM de proximité. Il s'agissait majoritairement d'hommes (67%), âgés de 63 ans [38-68], présentant un SDRA à Covid-19 (11/12). Aucune complication pendant le vol ou décès précoce (< 48h) n'a été relevé. Les rapports P/F avant et après transferts étaient comparables (174 et 191, p = 0,092). Les antécédents des patients (HTA, diabète, IMC > 30), l'association à une embolie pulmonaire, le pourcentage d'atteinte pulmonaire, la nécessité d'un support vasopresseur, le P/F avant départ ou encore le nombre de jour d'hospitalisation avant transfert, n’étaient pas des facteurs associés à la mortalité (p > 0,05). Le taux de mortalité était de 33 % (4/12).

## Discussion & Conclusion

Nous n'avons pas retrouvé de facteur prédictif d'aggravation de l’état du patient suite au transfert aérien. Le taux de décès était de 33 %, soit identique au taux national à cette même époque. Cette procédure mise en place durant l’été 2020 a permis de libérer des places de réanimation rapidement, conservant l'offre de soins locale, et sans perte de chance pour les patients transférés. Suite à ces retours favorables, des transferts transatlantiques ont pu être réalisés.

# Research article: Place de l'interculturalité dans les soins en Guyane : une étude qualitative

**Published:** 2022-06-30

**Authors:** Morgane EVENO, Blandine SOLIGNAT

**Affiliations:** 1 Centre hospitalier Andrée Rosemon, Cayenne, Guyane, France; 2 Institut de formation en soins infirmiers de Cayenne (IFSI), Guyane, France

## Contexte & Introduction

La Guyane française est un territoire multiculturel. Audelà des difficultés inhérentes à la barrière de la langue, les soignants sont confrontés à leurs propres représentations. L'objectif de cette étude était de mettre en évidence les compétences infirmières nécessaires à une prise en charge de qualité des patients de cultures différentes.

## Méthodologie

Une étude qualitative a été réalisée en 2021. Des entretiens semi-dirigés ont été conduits auprès d'infirmiers travaillant sur le territoire guyanais. La trame d'entretien comprenait des questions ouvertes portant sur leurs conditions d'exercice, l'influence de leurs propres représentations, la confrontation à l'interculturalité et le développement de compétences, ainsi que sur le recours à l'interdisciplinarité.

## Principaux résultats

Trois infirmiers ont participé à l’étude, travaillant sur Saint-Laurent-du-Maroni (urgences), Cayenne (libéral) et Maripasoula (CDPS). Les 3 infirmiers interrogés ont répondu être conscients de représentations culturelles pouvant influencer leurs prises en charge, l'exemple de la perception de la douleur suivant les origines était cité. Le facteur culturel majeur notifié comme obstacle aux soins était la barrière de la langue. Étaient aussi mis en avant les principes religieux ayant un impact sur les représentations de la santé et de la maladie en fonction des croyances. Les compétences à développer mises en évidence étaient :^1^ le savoir, développement des connaissances sur les cultures du territoire,^2^ le savoir-faire, capacité à maîtriser ses compétences professionnelles,^3^ le savoir-être, attitude et posture adaptées en tant que soignant. Au cours des entretiens, l'interdisciplinarité a été mise en avant, en particulier le recours à la médiation culturelle et à l'ethnopsychiatrie dans les prises en charge des patients.

## Conclusion

L'interculturalité est une notion essentielle en santé, en particulier en Guyane. De nombreux facteurs culturels, liés aux patients ainsi qu'aux soignants, influencent les prises en charge. Face à ces problématiques les soignants ont recours à leurs propres compétences relationnelles. Afin de promouvoir la qualité des soins, ces compétences pourraient être développées ainsi que le recours à l'interdisciplinarité, comme la médiation culturelle.

# Research article: Violences conjugales en Guyane : étude descriptive de juin 2019 à décembre 2020 à l'Unité médico-judiciaire du CH de Cayenne

**Published:** 2022-06-30

**Authors:** Margaux FOUILLET, Fabrice QUET, Karim HAMICHE, Angélique FRANCHI

**Affiliations:** 1 Service de médecine légale, Centre hospitalier de Cayenne, Guyane, France; 2 Département universitaire de médecine générale, Université des Antilles, Pointe-à-Pitre, Guadeloupe, France; 3 Centre d'investigation clinique Antilles Guyane (CIC INSERM 1424), Centre hospitalier de Cayenne, Guyane, France

## Contexte

Dans le monde, une femme sur trois est victime de violences physiques et/ou sexuelles de la part d'un partenaire intime. Peu de données existent sur les violences conjugales (VC) en Guyane. Les disparités sociales et les difficultés d'accès aux soins rendent la prise en charge des victimes de VC complexe à appréhender sur ce vaste territoire.

## Objectif

Décrire et analyser les caractéristiques sociodémographiques, cliniques et médico-légales associées aux victimes et aux situations de VC rencontrées à l'Unité médico-judiciaire (UMJ) du Centre hospitalier de Cayenne, Guyane, France

(CHC).

## Méthode

Étude observationnelle descriptive, rétrospective et monocentrique concernant des victimes, hommes et femmes de plus de 15 ans et 3 mois, ayant subi un ou plusieurs types de VC de la part d'un partenaire intime ou ex-partenaire intime et examinées sur réquisition à l'UMJ du CH de Cayenne entre le 1er juin 2019 et le 31 décembre 2020.

## Résultats principaux

466 certificats médicaux pour VC ont été analysés. 92,9 % des victimes sont des femmes. La moyenne d’âge est de 33 ans. 39,9 % des victimes sont étrangères et plus de la moitié ne travaillent pas. 31,5 % des victimes subissent des violences de la part d'un ex-partenaire intime. Les enfants sont témoins des violences dans 59,1 % des cas et victimes de maltraitance directe dans 18,8 % des cas. Dans 28,3 % des cas, la victime a déjà porté plainte antérieurement. Plus d'une victime sur deux a fait l'objet d'un signalement pour danger imminent (205/374) : séparation non consentie par l'auteur (42,8%), menaces de mort et/ou de suicide de l'auteur (58,8%), violences avec arme et/ou arme au domicile (35,0%), profil particulier de l'auteur (67,1%), vulnérabilité de la victime (33,2%), intensification des violences dans l'année précédente (52,9%), séquestration (15,2%), strangulation et/ou suffocation (46,5%), violences sexuelles (19,3%).

## Conclusion

Cette étude témoigne de la gravité des agressions subies et des facteurs de vulnérabilité existants : victime souvent jeune, étrangère, sans emploi, enceinte ou ayant des enfants. D'autres études sont nécessaires en population générale afin de pouvoir proposer des solutions pérennes et adaptées à l'ensemble du territoire.

# Research article: Création d'une équipe de coordination Covid en CDPS : retour d'expérience sur le déploiement de la vaccination Covid-19 dans les communes isolées de Guyane

**Published:** 2022-06-30

**Authors:** Estelle Jacoud, Sébastien Teissier, Véronique Servas, Amadine Papin, Frédéric Morandais, Fanny Gras, Céline Michaud, Mélanie Gaillet, Nicolas Vignier, Loïc Epelboin

## Introduction

En 2020, l’épidémie de Covid-19 a touché moins durement la Guyane que de nombreux territoires dans le monde. La proximité géographique du Brésil et le risque de dissémination du variant gamma ont nécessité la mise en place urgente d'une couverture vaccinale satisfaisante.

Loin du littoral et de ses 3 Centres hospitaliers vivent plus de 50 000 personnes isolées, multiculturelles et souvent précaires. Elles dépendent au plan sanitaire de 17 Centres délocalisés de prévention et de soins (CDPS). En février 2021, une équipe de « Coordination CDPS Covid » est créée afin d'organiser le déploiement de la vaccination et de la gestion du Covid au sein de ces territoires isolés.

## Méthode

Dès février 2021, deux médecins infectiologues à mi-temps, une infirmière coordinatrice, un logisticien et une secrétaire composent cette équipe. Elle travaille de manière coordonnée avec l’équipe de coordination des CDPS, l’Équipe mobile de santé publique en commune (EMSPEC) et les professionnels des CDPS.

## Résultats

Les tâches dédiées à cette équipe ont pris une ampleur inattendue. Certains éléments sont apparus comme essentiels pour permettre le déploiement de la vaccination anti-Covid au sein des territoires isolés. Le respect et le suivi de la chaîne du froid se sont avérés indispensables pour faire face aux modalités contraignantes de conservation des vaccins. Si certains CDPS sont accessibles par la route, d'autres sont très difficiles d'accès et nécessitent que les vaccins soient acheminés par voie aérienne (hélicoptère, avion) ou fluviale (pirogue).

Il a aussi fallu intégrer la dimension transfrontalière. Dès le début de la vaccination, une forte demande des citoyens de la rive brésilienne de l'Oyapock est apparue. Celle-ci a été motivée par la gravité de l’épidémie dans leur pays et le retard du déploiement vaccinal dans l'Amapá. La demande sur la rive surinamaise du Maroni de personnes travaillant dans l'orpaillage a été croissante au fur et à mesure du déploiement de la vaccination.

Afin de répondre aux différentes demandes, il a été nécessaire de redimensionner l'activité pour chaque CDPS en co-construisant avec les équipes au regard des besoins et de la capacité d'absorption de la charge. Ainsi, les vaccinations se sont déroulées au fil de l'eau ou sous forme d'actions massives. Bien évidemment, la mise en place d'une nouvelle activité a nécessité de recruter des professionnels médicaux et non médicaux, permettant de créer une équipe mobile de vaccination et un centre de vaccination sur Saint-Georges.

## Discussion & Conclusion

L’équipe de coordination a dû approfondir et adapter ses réflexions et ses stratégies en permanence afin d'offrir un accès équitable à la vaccination. L'augmentation progressive de l'acceptabilité vaccinale par la population, ainsi que la mise en place des vaccinations au fil de l'eau au sein des CDPS, nécessite un recadrage en ressources humaines des CDPS et un redimensionnement de l’équipe de coordination.

# Research article: De l’état des lieux à la construction de parcours de prise en charge des violences faites aux femmes : diagnostic situationnel dans l'Est guyanais

**Published:** 2022-06-30

**Authors:** Joliot PAULINE, Margaux BARBIER, Justine PEGUET-REVUZ, Italo MONTEIRO SOUSA, Jane Bordalo MILLER, Sophie RHODES

**Affiliations:** 1 Service des CDPS, Centre hospitalier Andrée Rosemon, Cayenne, Saint-Georges, Guyane, France; 2 Association IDsanté, Cayenne, Saint-Georges, Guyane, France; 3 Association DPAC Fronteira, Oiapoque, Brésil

## Contexte & Introduction

La Guyane enregistre le plus grand nombre de femmes victimes de violences conjugales en France, avec 10,4 femmes âgées de 20 ans ou plus pour 1 000 habitantes (Intérieur). Dans l'Est, les acteurs spécialisés sont rares. Dans ce contexte, un axe de lutte contre les violences faites aux femmes se développe depuis 2019 au sein du projet Oyapock Coopération Santé (OCS). Afin d'identifier plus finement les besoins, un diagnostic transfrontalier a été initié en 2020.

## Méthodologie

Une méthodologie essentiellement qualitative a été utilisée. Un travail de revue de la littérature et de recensement des acteurs nationaux, régionaux et locaux a d'abord été entrepris. Parallèlement, de mars 2020 à août 2021, 39 entretiens semi-directifs ont été menés auprès de professionnels impliqués dans la lutte contre les violences conjugales ou confrontés à ces violences dans leurs pratiques à Saint-Georges, Camopi et Oiapoque.

## Principaux résultats

Les trois communes font face à des problématiques similaires : isolement territorial, manque de structures ressources et de moyens, facteurs de vulnérabilité et précarité exacerbés, banalisation des violences. Les acteurs des 3 communes, qui partagent un sentiment d'impuissance face à l'ampleur du phénomène, ont exprimé des besoins analogues : formation, sensibilisation de la population, renforcement de la prise en charge et de l'accompagnement, développement du travail en réseau, création d'hébergements d'urgence. Des spécificités apparaissent cependant selon les pays ou les communes. Entre le Brésil et la France, prise en charge et réponse judiciaire varient significativement. Enfin, la banalisation et l'omerta sont contrastées entre les communes.

## Discussion & Conclusion

Ce diagnostic a entraîné à Saint-Georges la création de nouveaux dispositifs dans le but de définir des parcours de prise en charge des femmes victimes de violences : annuaire d'acteurs, coordination d'un réseau, cycles de formations, accompagnements personnalisés des femmes, recensement et partage des données sur les violences, staff hebdomadaire, création d'un espace ressource par IDsanté (association coordinatrice d'OCS) et d'outils de prévention originaux. Des perspectives de collaboration transfrontalière et d'actions à Camopi sont envisagées à moyen terme.

# Research article: Évaluation de l'efficacité et de l'impact de Malakit, intervention de lutte contre le paludisme sur le plateau des Guyanes, 2018-2020

**Published:** 2022-06-30

**Authors:** Yann LAMBERT, Muriel GALINDO, Alice SANNA, Louise MUTRICY, Irene JIMENO MAROTO, Mathieu NACHER, Antoine ADENIS, Stephen VREDEN, Martha SUAREZ-MUTIS, Maylis DOUINE

**Affiliations:** 1 Centre d'investigation clinique Antilles Guyane (CIC INSERM 1424), Centre hospitalier de Cayenne, Guyane, France; 2 Fondation pour l'avancement de la recherche scientifique au Suriname (SWOS), Paramaribo, Suriname; 3 Laboratoire de parasitologie, Fondation Oswaldo Cruz, Rio de Janeiro, Brésil

## Contexte

En Guyane, la population travaillant sur les sites d'orpaillage est la plus touchée par le paludisme. Sa mobilité importante sur le plateau des Guyanes et son recours fréquent à une automédication inadaptée font craindre l’émergence et la diffusion régionale de parasites résistants. En Guyane, un faisceau d'obstacles logistiques, règlementaires et sécuritaires limite le recours aux interventions habituelles auprès de cette population isolée.

## Méthodologie

Malakit est une intervention innovante reposant la réalisation du diagnostic et du traitement du paludisme par les orpailleurs eux-mêmes à l'aide d'un kit et d'une formation initiale assurée par des médiateurs. Fruit d'une collaboration internationale étroite, Malakit a été expérimenté entre avril 2018 et mars 2020 sur les frontières de la Guyane avec le Brésil et le Suriname. L’évaluation de l'efficacité et de l'impact de Malakit a reposé sur une méthodologie mixte : enquêtes transversales avant et après intervention, recueil de données longitudinales via un système d'information innovant, données de surveillance passive, entretiens qualitatifs semi-dirigés et modélisation mathématique.

## Principaux résultats

En deux ans, 3 733 participants ont été inclus dans Malakit, soit environ 30 % de la population cible. Les entretiens qualitatifs soulignent une perception positive de l'intervention par la communauté des orpailleurs. Le suivi longitudinal de 631 participants rapporte 72 % d'utilisation appropriée du kit, et l'absence d’événement indésirable grave. Les enquêtes avant/après Malakit auprès de 1 098 participants mesurent l'augmentation du recours à un test et un traitement adaptés (OR = 1,8), ainsi qu'une baisse de la prévalence de *Plasmodium* en PCR cohérente avec celle de l'incidence frontalière observée par les systèmes nationaux de surveillance épidémiologique. Une première estimation d'impact suggère que Malakit aurait permis d’éviter en moyenne 43 % de cas supplémentaires de paludisme sur la période 2018-2020.

## Discussion & Conclusion

Malakit montre que des personnes ayant un niveau d’éducation faible peuvent prendre en charge de manière autonome le diagnostic et le traitement d'un accès palustre. Cette nouvelle stratégie pourrait être transférée à d'autres régions faisant face à un contexte similaire.

# Research article: Évaluation du risque cardiovasculaire et de l'accès aux soins des agriculteurs en Guyane en 2018

**Published:** 2022-06-30

**Authors:** Amélie MARTINOT, Nadia THOMAS, Yoland GOVINDIN, Paul BROUSSE, Mathieu Nacher, Odette Nguyen VAN NAI, Cyril ROUSSEAU, Antoine ADENIS, Timothée BONIFAY

**Affiliations:** 1 Département universitaire de médecine générale, Université des Antilles, Pointe-à-Pitre, Guadeloupe, France; 2 Centre régional de coordination des dépistages des cancers, Cayenne, Guyane, France; 3 Mutualité sociale agricole, Caisse générale de sécurité sociale de Guyane, Cayenne, Guyane, France; 4 Pôle des Centres délocalisés de prévention et de soins, Centre hospitalier de Cayenne, Guyane, France; 5 Centre d'investigation clinique Antilles Guyane (CIC INSERM 1424), Centre hospitalier de Cayenne, Guyane, France

## Introduction

Au cours des dernières années, une détérioration globale de la santé des agriculteurs a été observée en France et la première cause de décès dans cette population reste les maladies cardiovasculaires. Il en est de même en Guyane où ces pathologies augmentent de manière préoccupante, et où peu de travaux de recherche ont été réalisés sur le monde agricole.

## Patients et méthodes

Étude transversale rétrospective analysant les données de l'action « Novembre vert » menée par la Mutualité sociale agricole (MSA) en 2018 en Guyane. Les critères d'inclusion : (i) être ayant droit ou exploitant agricole affilié à la MSA Guyane, et (ii) avoir participé à l'enquête (questionnaire, prise de paramètres, consultation médicale). L'objectif était d’évaluer le risque cardiovasculaire des agriculteurs guyanais et leur accès aux soins.

## Résultats

Un total de 603 agriculteurs a été inclus dans l’étude. Le sexe ratio H/F était de 1,6 et l’âge médian de 52 ans. Plus de 70 % des agriculteurs avaient un IMC ≥ 25 avec un surrisque d'obésité dans la population féminine. L'hypertension artérielle (HTA) touchait 53,1 % des agriculteurs et 80,1 % (n = 246/307) étaient dépistés lors de la campagne. Environ 13,5 % (n = 74/550) des agriculteurs étaient diabétiques. Sur ces bases, 27,0 % des participants étaient exposés à un risque cardiovasculaire haut ou très haut. Le risque cardiovasculaire était 3 fois plus présent chez les hommes (OR = 2,96; IC 95 % [1,92-4,56], p < 0,01). Les facteurs de risque les plus fréquents étaient : l'HTA et l'obésité chez les femmes, l’âge et l'HTA chez les hommes. L'accès aux soins est apparu bon mais avec des disparités, au total 89,6 % des agriculteurs accédaient à des soins bons ou intermédiaires. À noter un retard de dépistage des cancers comparable à celui de la métropole (59,9 % des personnes éligibles), des inégalités géographiques et une con-naissance partielle des aides sociales.

## Conclusion

Les résultats dépassent ceux attendus et constatent un risque cardiovasculaire élevé ou très élevé pour une grande partie des participants. Les prévalences de l'HTA (53,1%), de l'obésité (30,3%) et du diabète (13,5%) sont particulièrement préoccupantes. L’étude de l'accès aux soins est rassurante, mais révèle aussi des points faibles, notamment la couverture insuffisante des dépistages organisés. Il paraît essentiel de renforcer les actions auprès des agriculteurs guyanais, et une cohorte permettrait un suivi au long cours de leur santé.

# Research article: Causes et facteurs de risque de la mort fœtale in utero dans le bassin amazonien

**Published:** 2022-06-30

**Authors:** Meredith MATHIEU, Véronique LAMBERT, Benjamin MONIER, Gabriel CARLES, Najeh HCINI

**Affiliations:** 1 Service de gynécologie-obstétrique, Centre hospitalier de l'Ouest guyanais, Saint-Laurent-du-Maroni, Guyane, France; 2 Université de Guyane, Cayenne, Guyane, France

## Objectif

Notre objectif est de décrire l’épidémiologie des morts fœtales in utero dans l'Ouest de la Guyane et d'en évaluer les principales causes et les facteurs de risque.

## Méthodologie

Nous avons mené une étude descriptive rétrospective de janvier 2016 à décembre 2020. Nous avons extrait toutes les informations sur les morts fœtales in utero avec un âge gestationnel ≥ 20 semaines prises en charge par le Centre hospitalier de l'Ouest guyanais. Les interruptions de grossesse ont été exclues. Les informations concernant les antécédents médicaux, l'examen clinique, les résultats biologiques, l'histologie placentaire et l'examen autopsique ont été colligées pour déterminer l’étiologie du décès. Nous avons utilisé le système de classification INCODE *(Initial causes of fetal death evaluation)* pour répartir les causes de décès.

## Résultats

Au total, 276 fœtus parmi 269 accouchements de mort-nés ont été colligés. Le taux de mort fœtale variait entre 1,3 % et 2 %, avec une moyenne de 1,8 % sur 5 ans (276/14 869). Le suivi insuffisant de la grossesse ou l'absence de suivi (84/269, 31,2%), l'obésité ≥ 30 kg/m2 (54/269, 20%) ou la présence d'une prééclampsie (48/269, 17,8%) en ont été les principaux facteurs de risque. Selon la classification INCODE, les principales causes de mort fœtale étaient les complications obstétricales (34,1 %, 94/276), en particulier la très grande prématurité avant 26 semaines (50/94) et le décollement placentaire (30/94). Les infections materno-fœtales étaient fréquentes, notamment les maladies transmises par les moustiques (virus Zika, dengue et paludisme) et les agents infectieux réémergents, tels que la syphilis et les infections maternelles graves (2,2 %, 6/269). Au total, 24,6 % des décès fœtaux (68/276) sont restés inexpliqués. La précarité sociale et la fragilité du système de soins peuvent avoir un effet potentialisateur sur les issues défavorables de la grossesse.

## Discussion & Conclusion

Les troubles hypertensifs pendant la grossesse et la grande prématurité dans un contexte fréquent de précarité sont les principales causes de pertes fœtales dans l'Ouest de la Guyane. Une attention particulière doit être portée aux agents infectieux émergents et doit faire partie du bilan d'exploration des morts fœtales in utero.

# Research article: Épidémiologie et prise en charge des agressions par la faune sauvage aux urgences de Cayenne du 1^er^ janvier au 31 décembre 2019

**Published:** 2022-06-30

**Authors:** Jules MAURER, Rémi MUTRICY, Jean PUJO, Hatem KALLEL, Alexis FREMERY

**Affiliations:** 1 Service d'accueil des urgences, Centre hospitalier de Cayenne, Guyane, France; 2 Service de réanimation polyvalente, Centre hospitalier de Cayenne, Guyane, France

## Contexte

L'augmentation exponentielle de la population et l'extension des zones construites sur les forêts de Guyane multiplient les contacts entre les hommes et la faune sauvage. Ces espèces ont une réputation hostile pour l'homme en Amazonie. Bien qu'elles ne mènent que minoritairement à des conséquences graves, ces agressions restent des motifs de consultation fréquents en services d'urgences. À cela s'ajoutent certaines problématiques inhérentes à l’étendue du territoire, avec des populations isolées, qui soulèvent un certain nombre de défis à relever par le système de soins. L'objectif de ce travail est la description épidémiologique et la prise en charge médicale des victimes des agressions par la faune sauvage aux urgences de Cayenne.

## Matériels et méthodes

Étude observationnelle descriptive rétrospective du 1er janvier au 31 décembre 2019, incluant toutes les victimes d'agression par la faune sauvage prises en charge par le Service d'urgences de l'hôpital de Cayenne. Les données contextuelles des agressions, les données cliniques des patients ainsi que les données de la prise en charge des victimes ont été recueillies.

## Résultats

Sur l'année 2019, 402 patients agressés par la faune sauvage ont été inclus. Le ratio H/F était de 1,7 et l’âge médian était de 32 ans [16-49]. La classe animale la plus impliquée était celle des hyménoptères (26%), suivie des invertébrés non spécifiés (20%), des serpents (16%) et des scorpions (12%). La topographie des lésions concernait les membres inférieurs dans 43 % des cas. Des signes d'anaphylaxie ont été décrits chez 4 % des patients; 8 % des patients présentaient des signes neurologiques, et 8 % présentaient des signes digestifs. Un bilan biologique perturbé a été retrouvé chez 15 % des patients. À partir des données cliniques et biologiques, nous avons dénombré 92 patients graves ou à risque de s'aggraver, tous agressés par serpent (49%), insecte (42%) ou scorpion (9%). Aux urgences, 33 % des patients ont reçu des antalgiques, et 27 % ont été traités par antibiotiques, dont 92 % par amoxicilline – acide clavulanique. Les hospitalisations ont concerné 11 % des patients. Il n'y a pas eu de décès sur l'année.

## Conclusion

Parallèlement aux agressions par serpents et scorpions étudiées en détail ces dernières années, cette étude souligne l'aspect prépondérant aux urgences des agressions par hyménoptères. Les propriétés spécifiques des espèces en majorité responsables de ces agressions en font un sujet avec un certain nombre d'axes d'amélioration à envisager. Finalement, malgré une augmentation croissante des agressions par la faune au fil des années, il n'est à déplorer qu'une morbi-mortalité faible et stable dans le temps.

# Research article: Description épidémiologique et évaluation de la prise en charge des victimes de violences sexuelles au Centre hospitalier de Cayenne, Guyane, France

**Published:** 2022-06-30

**Authors:** Victoire MENSEAU, Jeanne CHARBONNIER, Angélique FRANCHI, Sihem OUAR, Mathieu NACHER, Alexis FREMERY

**Affiliations:** 1 Département universitaire de médecine générale, Université des Antilles, Pointe-à-Pitre, Guadeloupe, France; 2 Service des urgences, Centre hospitalier de Cayenne, Guyane, France; 3 Unité médico-judiciaire, Centre hospitalier de Cayenne, Guyane, France; 4 Centre d'investigation clinique Antilles Guyane (CIC INSERM 1424), Centre hospitalier de Cayenne, Guyane, France

## Introduction

Les violences sexuelles sont un enjeu majeur de santé publique. En Guyane, le sentiment d'insécurité est plus important que sur le reste du territoire français. Les violences sexuelles sont un motif important de consultation en médecine légale et d'urgence. L'enjeu est une prise en charge dans les 72 heures suivant l'agression, notamment sur les plans médico-légal ou infectieux. Des données descriptives manquent sur le territoire guyanais. Les objectifs de l’étude étaient de décrire la population des victimes d'agression sexuelle reçues au Centre hospitalier de Cayenne, Guyane, France et d’évaluer leur prise en charge.

## Matériels et méthodes

Une étude rétrospective descriptive monocentrique a été menée entre le 1er janvier 2019 et le 31 décembre 2020. Tous les patients ayant consulté pour une agression sexuelle à l'Unité médico-judiciaire (UMJ), au Service d'accueil des urgences (SAU) ou à l'Hôpital de jour adulte (HDJA) ont été inclus.

## Résultats

Sur cette période, 400 victimes ont consulté à l'UMJ pour agression sexuelle. La majorité des victimes étaient des femmes (86,8%). La médiane d’âge était de 13 ans [8-17,5]. L'agresseur était majoritairement de sexe masculin (99,2%), le plus souvent connu de la victime (86,7%) et issu du cercle familial (39,8%). Les suspicions d'agression représentaient 18,8 % des réquisitions de l'UMJ et concernaient principalement les victimes de moins de 10 ans (82,7%). L'agression la plus fréquente chez les femmes était la pénétration pénio-vaginale (82,2%), et pénio-anale (76,9%) chez les hommes. Le délai de consultation était supérieur à 72 h dans 60,6 % des cas. Une consultation psychologique était recommandée pour 61,8 % des victimes. Nous avons comparé ces données aux 139 passages des urgences pour ce motif et 31 passages à l'HDJA. Nous avons comptabilisé 73,4 % des victimes des urgences vues à l'UMJ. Seize victimes ont été hospitalisées. Parmi les victimes de l'UMJ adressées à l'HDJA, seules 11,3 % s'y sont effectivement rendues.

## Conclusion

Ce travail a permis d'identifier une population victime, féminine, jeune, le plus souvent agressée par une personne connue d'elle et de sexe masculin. Le délai de consultation était majoritairement supérieur à 72 heures. Lors du suivi de ces patients, nous avons relevé que les victimes prises en charge à l'UMJ et adressées à l'HDJA ne s'y sont pour la plupart pas rendues. Au regard de cette étude, un certain nombre d'axes d'amélioration de prévention et de prise en charge ont été proposés.

# Research article: Résultat d'une année d'activité (2017) d'une infirmière dédiée au dépistage du VIH par TROD aux urgences de Cayenne

**Published:** 2022-06-30

**Authors:** Vanessa MOREL, Aude LUCARELLI, Achille GAUDINOT, Leila ADRIOUCH, Émilie Gaubert-MARÉCHAL, Philippe ABBOUD, Pierre COUPPIÉ, Mathieu NACHER, Félix DJOSSOU, Loïc EPELBOIN

**Affiliations:** 1 Centre hospitalier Andrée Rosemon, Cayenne, Guyane, France; 2 Centre d'investigation clinique Antilles Guyane (CIC INSERM 1424), Centre hospitalier de Cayenne, Guyane, France

## Contexte & Introduction

En Guyane, où la prévalence est la plus élevée de France (> 1%), on estime entre 500 à 600 le nombre de personnes ignorant leur séropositivité sur une population de plus de 250 000 habitants. Aux urgences du Centre hospitalier de Cayenne, Guyane, France, une infirmière dédiée est en charge de l'activité de dépistage du VIH.

## Méthodologie

L’étude s'est déroulée du 1er janvier au 31 décembre 2017, parmi les patients de plus de 18 ans consultant aux urgences pour tout motif. L'IDE, présente de 8 h à 15 h du lundi au vendredi, propose aux patients de réaliser un dépistage du VIH par un TROD (test rapide d'orientation diagnostique) après un entretien personnalisé. Sont exclus les patients de la filière psychiatrique, les mineurs, les patients ne pouvant réaliser l'entretien et ceux accompagnés par les forces de l'ordre.

## Résultats

Mille quatre-vingt-dix-huit patients ont été abordés et 688 ont été dépistés. Le sexe ratio H/F des personnes interrogées était de 1,2. Parmi eux, 80 % avaient déjà fait un dépistage au cours de leur vie, 64 % au cours des 5 dernières années et 36 % au cours des 12 derniers mois. Parmi les 217 patients (24%) ayant refusé le TROD, les principales explications fournies étaient l'absence alléguée de prise de risque et la réalisation récente d'un test. Cependant, 13 % d'entre eux n'avaient jamais eu recours à un test et 35 % avaient un test de plus d'un an. La principale raison évoquée d'accepter le TROD était de réaliser un contrôle de routine (68%). Une prise de risque dans le passé était rarement invoquée (12%). 51 % avaient réalisé un dépistage au cours des cinq dernières années et 37 % avaient un test < 1 an. Une grande part de la population interrogée pensait que le dépistage VIH était réalisé systématiquement lors de toute prise de sang. Sur ces 688 TROD réalisées, 11 (1,6%) sont revenus positifs, parmi lesquels 6 découvertes réelles et 5 déjà connus (1 non observant, 1 déni, 1 perdu de vue, 2 n'ayant pas compris l'objet de l'entretien). Les personnes reconvoquées en consultation se sont présentées au rendez-vous, sauf celle qui était déjà dans le déni de sa pathologie.

## Discussion & Conclusion

Comparée à l’étude de référence réalisée dans 29 services d'urgences d’Île-de-France en 2009 et publiée dans *Archives of Internal Medicine,* l'acceptabilité du test a été bonne aux urgences de Cayenne. L'impact de l’étude à Cayenne a été significativement plus important que celui en région parisienne qui avait considéré comme très modeste, 18 sur 12 574 personnes testées positives ne connaissant pas leur séropositivité (0,14%) *vs* 6 sur 688 (0,87%) (p = 0,001; OR 6,1; IC 95 % [2,0-16,1]). Lors de l’étude parisienne, les personnes dépistées appartenaient à des populations à risque (hommes homosexuels et Africains subsahariens), le dépistage ciblé étant privilégié, tandis que les patients positifs de notre étude étaient des patients tout venant. Le dépistage généralisé en Guyane par une infirmière dédiée semble donc une action pertinente. En 2018, la mission de cette infirmière évolue, avec extension du dépistage par TROD à tous les patients hospitalisés au sein du Centre hospitalier de Cayenne, Guyane, France et la création d'une équipe mobile de dépistage.

# Research article: Enquête exploratoire sur la barrière de la langue dans les soins en Guyane en juillet 2021

**Published:** 2022-06-30

**Authors:** Sindiami MORISSEAU, Malika BOUMGHAR

**Affiliations:** 1 Centre hospitalier de l'Ouest guyanais, Guyane, France; 2 Centre hospitalier Andrée Rosemon, Cayenne, Guyane, France; 3 Institut de formation en soins infirmiers de Cayenne (I.F.S.I.)

## Contexte & Introduction

En Guyane, la barrière de la langue est fréquente lors de la prise en charge des patients non francophones. L'objectif de cette enquête était de mettre en lumière les difficultés et les outils/modes de communication mis en place par les soignants et les patients afin d'améliorer nos pratiques.

## Méthodologie

Nous avons effectué des entretiens semi-directifs appuyés par un questionnaire de 4 questions liées à la barrière de la langue : 1) Avez-vous déjà été confronté à des situations de soins compliquées du fait de la barrière de la langue ? 2) Comment communiquer avec un patient non francophone et comment obtenir son consentement ? 3) Quels outils utilisez-vous pour réaliser vos soins techniques et relationnels ? 4) Comment évaluez-vous le ressenti des patients après un soin ? Étaient éligibles aux questionnaires les infirmières IDE et les médiateurs culturels exerçant en Guyane.

## Principaux résultats

Quatre professionnels ont été interrogés (1 IDE diabétologie, 1 IDE de chirurgie, 1 IDE d'HAD et 1 médiateur culturel de la Croix-Rouge). Question 1 : L'ensemble des soignants étaient confrontés à la barrière de la langue. Certaines langues posaient plus de problèmes : « On peut se débrouiller en portugais, en anglais, en espagnol, mais en taki-taki quand la traductrice n'est pas présente, c'est vraiment compliqué ». Question 2 : En l'absence de médiateurs, les soignants utilisaient des outils de « débrouillage » tels que le mime, le téléphone portable avec traducteur, l'appel d'un membre de la famille. Ce qui avait pour conséquences d'augmenter le temps de soin. L'obtention d'un consentement éclairé était difficile et ces situations étaient mal vécues par les soignants : « J'ai parfois l'impression de n'avoir rien fait pour le patient. » Question 3 : La douleur était évaluée à l'aide de scores habituellement réservés aux patients de pédiatrie ou gériatrie (échelle des visages ou score Algo+). Question 4 : L’évaluation du ressenti semblait difficile pour l'ensemble des soignants. Le faciès ou la posture pouvaient être des éléments d'orientation.

## Conclusion

La barrière de la langue est une problématique quotidienne dans l'exercice du soin en Guyane. Elle semble être un obstacle à un consentement éclairé, et ces situations sont parfois mal vécues par les soignants.

# Research article: Comportements à risque sexuel et addictif, et infections sexuellement transmissibles chez les orpailleurs illégaux en Guyane : une étude observationnelle multicentrique

**Published:** 2022-06-30

**Authors:** Louise MUTRICY-HUREAU, Amandine PISONI, Martha SUAREZ-MUTIS, Amanda FIGUEIRA DA SILVA, Yann LAMBERT, Pauline MESPOULHE, Audrey GODIN, Marie-Claire PARRIAULT, Astrid Van Melle, Émilie Mosnier, Mélanie GAILLET, Céline MICHAUD, Roxane SCHAUB, Muriel Galindo, Antoine ADENIS, Mathieu NACHER, Stephen VREDEN, Édouard TUAILLON, Maylis DOUINE

**Affiliations:** 1 Centre d'investigation clinique Antilles Guyane (CIC INSERM 1424), Centre hospitalier de Cayenne, Guyane, France; 2 UMR 1058 INSERM/EFS/Université de Montpellier, Pathogénie et contrôle des infections chroniques, CHU de Montpellier, Service de bactériologie-virologie, Montpellier, France; 3 Fondation Oswaldo Cruz (Fiocruz), Laboratoire des maladies parasitaires, Rio de Janeiro, Brésil; 4 CRB Amazonie (Centre de ressources biologiques), Centre hospitalier de Cayenne, Guyane, France; 5 Unité des maladies infectieuses et tropicales (UMIT), Centre hospitalier de Cayenne, Guyane, France; 6 Université Aix-Marseille, INSERM, IRD, SESSTIM (Sciences économiques et sociales, systèmes de santé et informatique médicale), Marseille, France; 7 Centres délocalisés de prévention et de soins (CDPS), Centre hospitalier de Cayenne, Guyane, France; 8 Tropical Biome and Immunophysiopathology (TBIP), Université de Guyane, Université de Lille, CNRS, INSERM, Institut Pasteur de Lille, U1019-UMR9017-CIIL Centre d'infection et d'immunité de Lille, France; 9 Fondation pour l'avancement de la recherche scientifique au Suriname (SWOS), Paramaribo, Suriname

## Contexte

Les représentations courantes du monde de l'orpaillage – notamment illégal – sont généralement négatives : l'activité évoque des images de trafic de drogue, d'exploitation humaine, le commerce sexuel, la destruction de l'environnement et les maladies infectieuses, notamment les infections sexuellement transmissibles (IST). L'objectif du présent article est de décrire les niveaux de comportements à risque tels que le sexe transactionnel, les partenaires sexuels multiples et la fréquence de l'utilisation du préservatif, la consommation de substances addictives, ainsi que la prévalence des IST parmi la population des mineurs d'or illégaux en Guyane, une entité française d'outre-mer en Amazonie, afin d'orienter les interventions potentielles.

## Méthode

Une étude transversale observationnelle multicentrique a été réalisée d'octobre à décembre 2019 le long des deux frontières de la Guyane avec le Suriname et le Brésil, sur des sites de repos utilisés par les mineurs.

## Résultats

Parmi les 499 participants, les rapports sexuels transactionnels étaient très prévalents, déclarés par 33,5 % des hommes et 8,4 % des femmes. Les préservatifs étaient plus fréquemment utilisés pour les rapports transactionnels qu'avec un partenaire non commercial (93,4 % contre 42,1%). Plus de femmes ont été testées pour le VIH que les hommes (91,1 % contre 55,2%). La consommation excessive d'alcool (57,3%) et le tabagisme (41,2%) sont très fréquents, mais la consommation de cocaïne ou de crack est faible (1,2%), ce qui réfute notre hypothèse initiale. Les consommateurs d'alcool avaient plus de partenaires sexuels et ont déclaré utiliser le préservatif plus fréquemment. La prévalence du VIH, du VHC, du VHB et de la syphilis était respectivement de 0,5 % (IC 95 % : 0,1-2,1), 2,1 % (IC 95 % : 0,7-3,6), 1,6 % (IC 95 % : 0,3-2,8) et 12,4 % (IC 95 % : 9,0-15,7), ce qui est plus élevé que dans la population locale, en particulier pour la syphilis.

## Conclusion

Cette étude documente pour la première fois les comportements à risque des chercheurs d'or en Guyane. Bien que le niveau d'utilisation du préservatif soit élevé, la prévalence des IST combinée au taux élevé de rapports sexuels transactionnels devrait inciter à la prudence. Le taux élevé de rapports sexuels transactionnels devrait inciter à augmenter la prévention et de dépistage, notamment par des tests rapides, étant donné la mobilité de la population concernée.

# Research article: Épidémiologie des cas de tuberculose maladie au Centre pénitentiaire de Guyane

**Published:** 2022-06-30

**Authors:** Niaux MOÏSE, Mathilde BOUTROU, Mathieu NACHER, Félix DJOSSOU, Marie DANIEL, Vanessa SCHIEMSKY, Florence HUBER, Timothée BONIFAY

**Affiliations:** 1 Unité sanitaire en milieu pénitentiaire, Centre hospitalier de Cayenne, Guyane, France; 2 Département universitaire de médecine générale, Université des Antilles, Pointe-à-Pitre, Guadeloupe, France; 3 Unité des maladies infectieuses et tropicales, Centre hospitalier de Cayenne, Guyane, France; 4 Centre d'investigation clinique Antilles Guyane (CIC INSERM 1424), Centre hospitalier de Cayenne, Guyane, France; 5 Centre de lutte anti-tuberculeuse, Croix-Rouge française de Guyane, Cayenne, Guyane, France

## Introduction

La tuberculose est l'une des 10 premières causes de mortalité dans le monde. Les personnes détenues en Guyane représentent une population particulièrement à risque, cumulant les facteurs de risque. À ce jour, aucune étude n'a étudié cette population particulière concernant la tuberculose maladie (TM). L'objectif de cette étude était de décrire la population ayant eu une TM au Centre pénitentiaire de Guyane (CPG) ou dans les deux ans suivant la sortie d'incarcération, entre 2008 et 2020.

## Méthodes

Étude de cohorte rétrospective monocentrique sur cas de tuberculose maladie au CPG de 2008 à 2020. Les critères d'inclusion : être incarcéré sur la période de l’étude et avoir déclaré une TM. Les critères d'exclusion : être diagnostiqué hors de la période d’étude, les erreurs diagnostiques, les diagnostics précédant toute incarcération et les cas cumulant trop de données manquantes.

## Résultats

À partir des 36 cas de TM étudiés, un profil de personnes à risques a pu être dessiné, reprenant en partie les facteurs de risque de TM déjà connus (origine étrangère, précarité, consommation de toxiques, etc.). Il apparaît une forte incidence avec 263 cas/100 000 entrées en prison, plus élevée qu'ailleurs en France et plus comparable avec les incidences brésiliennes. On remarque une part importante des patients déclarant une TM dans l'année suivant leur libération. Cependant, le dépistage systématique à l'entrée en incarcération semble efficace, avec peu de signes de circulation intra-carcérale de TM.

## Discussion

Cette étude est limitée par la taille de sa population, la possibilité de sous-évaluation des TM en raison de difficultés d'identitovigilance dans cette population et d'une possible sous-déclaration des cas.

Une poursuite du dépistage à l'entrée, en association avec un dépistage annuel radiologique et clinique ainsi qu'un suivi renforcé à la sortie semblent indiqués pour améliorer la prise en charge des patients.

# Research article: Équipe mobile WASH en situation d’épidémie de Covid-19

**Published:** 2022-06-30

**Authors:** Margot OBERLIS, Étiennette RENOUD-GRAPPIN, Émilie SEVESTRE, Jean-Luc BAUZA, Attaher Souleymane GOURGOUDOU, Charlène COCHET, Solène WIEDNER-PAPIN, Karl KPOSSOU, Mélanie GAILLET, Florence HUBER

**Affiliations:** 1 Croix-Rouge française, Cayenne, Guyane, France; 2 Pôle des Centres délocalisés de prévention et de soins (CDPS), Centre hospitalier de Cayenne, Guyane, France; 3 Agence régionale de santé, Cayenne, Guyane, France; 4 Guyane Promo Santé, Instance régionale en éducation et promotion de la santé, Cayenne, France

La Guyane est confrontée depuis deux ans à la circulation du virus COVID-19 et traverse des vagues épidémiques successives mettant à mal son système économique et sanitaire. Exacerbées par cette crise sanitaire, les conditions de vie des personnes en situation de précarité s'altèrent. Maintenir un accès à l'eau et une gestion de la ressource est un des points clés de la réponse à la gestion de la crise.

Sollicitée par l'Agence régionale de santé (ARS) dès septembre 2020, la Croix-Rouge française déploie le projet WASH *(Water Sanitation Hygiene,* ou Eau Hygiène Assainissement) et accompagne les habitants des zones informelles et des villages isolés dans la lutte contre la propagation du Covid-19 et d'autres maladies à risque épidémique (certaines maladies hydriques, maladies à transmission vectorielle).

Projet en promotion de la santé, le projet WASH propose une approche innovante en aidant les habitants des zones d'intervention à mieux se prémunir contre différentes pathologies inhérentes au territoire, visant à réduire les risques sanitaires à des niveaux individuels et collectifs.

Cofinancé par le Fonds social européen, la DGCOPOP et l'ARS, le projet WASH intervient avec de nombreux partenaires sur l'ensemble du territoire.

L’équipe projet, composée d'une cheffe de projet, de deux coordinatrices terrain et de 11 agents de santé publique issus de groupes culturels variés, intervient au sein des quartiers d'habitat spontané des communes littorales et accompagne les habitants des villages isolés des communes de l'intérieur.

L'approche du projet WASH repose sur l'empowerment des populations et sollicite la mobilisation sociale pour la mise en place d'actions collectives par et pour les habitants.

Des maraudes de sensibilisation et des animations collectives sont proposées, visant à les familiariser avec différentes thématiques du projet. La présence régulière de l’équipe a permis d’établir un solide lien de confiance et d'accompagner les personnes souhaitant structurer des comités, garants de la pérennité des actions de prévention et des interventions collectives mises en œuvre.

Fin janvier 2022, 270 sensibilisations sur les gestes barrières et la vaccination contre la Covid-19 ont été réalisées sur l'ensemble du territoire, soit 8 800 personnes informées.

Quatre-cent-cinquante-trois actions de maraudes et d'animations collectives ont également permis de sensibiliser 10 500 personnes sur le transport, le stockage et la potabilisation de l'eau, la gestion des déchets ainsi que sur les maladies entériques et vectorielles. Cinq mille kits hygiène et 230 jerricans ont été distribués aux personnes les plus nécessiteuses durant cette crise sanitaire. 10 comités de quartier ont été accompagnés, de leur structuration à la mise en œuvre d'actions collectives au sein des quartiers d'habitats spontanés.

Forte de ces 18 mois d'intervention, l’équipe WASH a fait émerger des besoins permanents d'accès à l'information sanitaire de base et témoigne d'une importante capacité d'agir des populations.

Désormais, le défi consiste à passer de la notion d'urgence à une véritable politique en promotion de la santé sur les thématiques EHA en proposant une approche transversale, adaptée aux différents contextes du territoire et renforçant les populations dans leur prise en charge en promouvant une approche communautaire en santé.

# Research article: Risque de rechute de toxoplasmose cérébrale en fonction de l’évolution de l'imagerie : une étude rétrospective en Guyane de 2008 à 2020

**Published:** 2022-06-30

**Authors:** Sandrine PEUGNY, Harold CHIRON, Balthazar NTAB, Pierre COUPPIÉ, Magalie DEMAR, Magaly ZAPPA, Follikoe YAO HOUNSI, Hatem KALLEL, Milko SOBESKY, Mathieu NACHER, Loïc EPELBOIN, Félix DJOSSOU

**Affiliations:** 1 Unité des maladies infectieuses et tropicales, Centre hospitalier de Cayenne, Guyane, France; 2 Service de radiologie, Centre hospitalier de Cayenne, Guyane, France; 3 Département d'information médicale, Centre hospitalier de l'Ouest guyanais, Saint-Laurent-du-Maroni, Guyane; 4 Service de dermatologie-vénérologie, Centre hospitalier de Cayenne, Guyane, France; 5 Laboratoire hospitalo-universitaire de parasitologie-mycologie, Centre hospitalier de Cayenne, Guyane, France; 6 Service de radiologie, Centre hospitalier de l'Ouest guyanais, Saint-Laurent-du-Maroni, Guyane, France; 7 Service des soins critiques, Centre hospitalier de Cayenne, Guyane, France; 8 Département d'information médicale, Centre hospitalier de Cayenne, Guyane, France; 9 CIC INSERM 1424, Centre hospitalier de Cayenne, Guyane, France

## Introduction

La toxoplasmose cérébrale (TC) est la deuxième infection opportuniste la plus fréquente en Guyane. Des études menées avant l'arrivée des trithérapies antirétrovirales ont décrit des caractéristiques d'imagerie prédictives de rechute de la TC. Cette étude compare les images cérébrales avant et après traitement *anti-Toxoplasma* chez les personnes vivant avec le virus de l'immunodéficience humaine (PVVIH), et évalue les caractéristiques radiologiques pouvant prédire le risque de rechute de TC.

## Méthodes

Une étude de cohorte rétrospective a été menée dans deux hôpitaux de Guyane. Les PVVIH hospitalisés pour TC et recevant plus de 3 semaines de traitement *anti-Toxoplasma* ont été inclus. Les imageries cérébrales réalisées entre 21 et 90 jours après le début du traitement ont été revues par un radiologue de l'hôpital de Cayenne, en aveugle des données cliniques et biologiques. Le critère de jugement principal était la rechute de TC dans les 12 mois suivant un traitement *anti-Toxoplasma* adéquat.

## Résultats

Entre janvier 2008 et décembre 2020, 40 épisodes de TC ont été inclus. Le délai moyen entre l'imagerie pré- et post-traitement était de 45 ± 13 jours. Une régression complète des lésions de TC a été obtenue sur 7,5 % des images post-thérapeutiques (n = 3). Une prise de contraste persistait sur 87,5 % des imageries post-thérapeutiques (n = 35). Sept ont rechuté, en moyenne 6,8 ± 4,4 mois après le diagnostic de TC. Sur les 7 ayant rechuté dans les 12 mois, 5 avaient une localisation dans les noyaux gris centraux et/ou le thalamus. Le nombre, la taille et la persistance du rehaussement après le traitement ne différaient pas entre les groupes récidive et absence de récidive.

## Conclusion

Le rehaussement lésionnel, persistant sur 35/40 images post-thérapeutiques, n’était pas un facteur prédictif de rechute de TC. La localisation profonde des lésions de TC semble être associée à un taux de rechute plus fréquent à 12 mois, sans que cela soit statistiquement différent.

# Research article: Étude observationnelle BECATOUR concernant les AVC dans 3 villes françaises : Besançon, Cayenne, Tours

**Published:** 2022-06-30

**Authors:** Johanna RHEIN, Mathieu NACHER, Thierry MOULIN, Bertrand de TOFFOL

**Affiliations:** 1 Service de neurologie, Centre hospitalier de Cayenne, Guyane, France; 2 Centre d'investigation clinique Antilles Guyane (CIC INSERM 1424), Centre hospitalier de Cayenne, Guyane, France; 3 Unité de soins intensifs neurovasculaires, Département de neurologie, Centre hospitalier universitaire de Besançon, France

## Contexte

L'AVC est un enjeu majeur de santé publique. Son épidémiologie est encore mal connue en Guyane.

## Méthode

Nous avons réalisé une étude observationnelle prospective comprenant 100 patients consécutifs hospitalisés pour AVC à Cayenne, Tours et Besançon. Nous avons comparé leurs antécédents, leurs facteurs de risque cardio-neuro-vasculaire, leurs scores de Rankin et NIHSS, leurs traitements d'entrée, la prise en charge en phase aiguë, la typologie de leur AVC, leur durée d'hospitalisation, le mécanisme de leur AVC selon la classification TOAST, leurs traitements de sortie, leur mode de sortie.

## Résultats

En Guyane, l’âge moyen des patients est inférieur de 7 ans (62,5 ans), les patients sont plus fréquemment atteints d'hypertension artérielle (75/100) et de diabète (31/100). Les infarctus d'origine lacunaire y sont sur-représentés (13/100), et les infarctus d'origine cardio-embolique sous-représentés (12/100). Les scores NIHSS d'entrée et de sortie sont similaires entre Guyane et France métropolitaine. La prise en charge en phase aiguë est très différente : le taux de patients thrombolysés (9/100) est 3 à 4 fois moindre, la thrombectomie n'est pas disponible. Les patients sont moins nombreux à être transférés en centre de rééducation (14/100), plus de patients étant transférés en hospitalisation à domicile (6/100).

## Discussion

Les infarctus cérébraux éligibles à une thrombectomie sont sur-représentés dans les deux CHU de France métropolitaine, car les patients venant d'hôpitaux de périphérie y sont parfois transférés dans ce but. Ce biais induit une sur-représentation des infarctus plus sévères, et probablement une sur-représentation des AVC d'origine cardio-embolique. Les infarctus d'origine indéterminée sont plus nombreux en Guyane, car les patients sortent souvent de l'hôpital avec un bilan cardiologique incomplet.

## Conclusion

Le profil des patients admis pour AVC est différent en Guyane par rapport à la France métropolitaine. La mise en place d'une unité de soins intensifs neurovasculaires et d'une campagne d'information sur les symptômes de l'AVC permettrait une meilleure prise en charge et un plus grand nombre de thrombolyses.

# Research article: Connaissance, attitude et pratique des chiroptérologues vis-à-vis de la rage en Amazonie française

**Published:** 2022-06-30

**Authors:** Brigitte ROMAN-LAVERDURE, Lucie VIAVANT, Philippe ABBOUD, Gaëlle WALTER, Alessia MELZANI, Émilie MOSNIER, Vincent RUFRAY, Guillaume VESIN, Félix DJOSSOU, Loïc EPELBOIN

**Affiliations:** 1 Unité des maladies infectieuses et tropicales, Centre de traitement antirabique, Centre hospitalier de Cayenne, Guyane, France; 2 Centres délocalisés de prévention et de soins, Centre hospitalier de Cayenne, Guyane, France; 3 Groupe Chiroptères de Guyane, Cayenne

## Contexte & Introduction

En Guyane, les deux principales catégories d'animaux responsables de morsures à risque de transmission du virus de la rage et aboutissant à une prise en charge médicalisée sont les chiens et les chiroptères, en particulier l'espèce hématophage *Desmodus rotondus* ou vampire. Plus de 100 espèces de chauves-souris existent en Guyane, et toutes sont potentiellement porteuses du virus, ce qui expose les scientifiques et naturalistes étudiant les chauves-souris et les chiroptérologues à un risque répété lors des captures à visée scientifique. L'objectif primaire de cette étude était d’évaluer les connaissances de ces professionnels vis-à-vis du risque rabique et de sa prévention. L'objectif secondaire était d’évaluer leurs besoins en formation et informations.

## Méthodologie

Étude prospective descriptive sur la base d'un auto-questionnaire mis en ligne (Sondage Online) réalisé sur la période d'un mois (du 6 octobre au 6 novembre 2017) et adressé aux 60 membres de la liste de diffusion « chiroguyane ». Les données en rapport avec leur pratique, leurs connaissances de la maladie, de son mode de transmission, du traitement, de la vaccination antirabique et du suivi sérologique ont été analysées.

## Principaux résultats

Vingt personnes ont répondu au questionnaire (33 % de la liste de diffu-sion), dont 19 jusqu'au bout. 65 % étaient des hommes, 100 % originaires d'Europe, 45 % de plus de 40 ans. 50 % étaient en contact direct avec des chauves-souris au moins une fois par mois et 20 % plusieurs fois par semaine. 37 % manipulaient à mains nues. 95 % savaient que l'agent de la rage est un virus, 75 % qu'il touche le cerveau et 35 % les nerfs. 95 % savaient qu'il existe un traitement post-exposition, vaccin pour 54 % d'entre eux et immunoglobulines pour 27 %. 84 % savaient que la maladie est systématiquement mortelle une fois déclarée. 79 % et 89 % ne savaient pas que le génotype 1 RABV était responsable de la rage en Guyane chez les chiens et les chauves-souris. 59 % identifiaient les chauves-souris comme réservoir de la rage en Guyane et 33 % comme vecteur principal, 28 % pour les chiens. Seules 2 personnes ignoraient qu'il y avait déjà eu un cas de rage en Guyane (11%). 63 % savaient que la vaccination pré-exposition était recommandée chez les chiroptérologues et avaient une vaccination antitétanique de moins de 10 ans. 79 % avaient eu un protocole antirabique complet et datant de moins de 10 ans. 56 % d'entre eux avaient fait au moins un contrôle d'anticorps. 63 % savaient qu'une consultation au Centre de traitement antirabique était nécessaire à chaque nouvelle exposition, et 90 % pensaient que cette vaccination était nécessaire à leur activité. 63 % pensaient être insuffisamment informés et 79 % souhaitaient plus d'informations.

## Discussion & Conclusion

Les chiroptérologues de Guyane semblent assez bien informés sur la rage et le risque inhérent à leur activité, en contraste avec l'importance des prises de risque notamment lors des manipulations trop fréquemment à mains nues. Les connaissances sur les possibilités thérapeutiques sont médiocres et la couverture vaccinale déclarée insuffisante, sans parler du contrôle sérologique. Les demandes et besoins en informations concernant la prise en charge post-exposition du risque rabique et le suivi sérologique sont importants et pourront être satisfaits via des informations en ligne, groupes de diffusion et Facebook, mais aussi par l'organisation de for-mations dans un cadre associatif, les personnes concernées provenant de milieux professionnels hétérogènes.

# Research article: Évidence sérologique de la circulation de la fièvre Q dans les élevages de ruminants en Guyane de 2015 à 2017

**Published:** 2022-06-30

**Authors:** Mona SAOUT, Thibaut LURIER, Loïc EPELBOIN, Xavier BAUDRIMONT, Denis BLANCHET, Élodie ROUSSET, Magalie DEMAR

**Affiliations:** 1 Tropical Biome and Immunophysiopathology (TBIP), Université de Guyane, Cayenne, Guyane, France; 2 Université Clermont Auvergne, INRAE, VetAgro Sup, UMR EPIA, F-63122 Saint-Genès-Champanelle, France. Université de Lyon, INRAE, VetAgro Sup, UMR EPIA, F-69280 Marcy l'Etoile, France. Université de Lyon, INRAE, VetAgro Sup, USC 1233 UR RS2GP, 69280 Marcy-l'Etoile, France; 3 Université de Lille, CNRS, INSERM, Institut Pasteur de Lille, U1019-UMR9017-CIIL Centre d'infection et d'immunité de Lille, France; 4 Centre hospitalier de Cayenne, Guyane, France; 5 Unité Santé et protection animales et végétales environnement, Direction de l'environnement, de l'agriculture, de l'alimentation et de la forêt (DEAAF), DGTM Guyane, Cayenne, Guyane, France; 6 ANSES (Agence nationale de sécurité sanitaire de l'alimentation, de l'environnement et du travail), Laboratoire de Sophia Antipolis, Unité fièvre Q animale, Laboratoire national de référence sur la fièvre Q, Sophia Antipolis, France

## Contexte & Introduction

La fièvre Q est une zoonose causée par la bactérie *Coxiella burnetii,* de répartition mondiale et dont le taux d'incidence chez l'homme en Guyane est le plus élevé au monde. L’épidémiologie de cette maladie au niveau mondial est liée aux élevages de bovins, caprins et ovins. Tout type de mammifère est potentiellement hôte et excréteur de *C. burnetii,* bactérie persistante dans l'environnement. La circulation du pathogène n'a que rarement et ponctuellement été mise en évidence jusqu’à ce jour en Guyane. Les interrogations concernant les réservoirs animaux de la fièvre Q en Guyane sont rémanentes.

## Méthodologie

Une étude de séroprévalence de la fièvre Q a été menée sur 1 228 sérums provenant de 109 élevages bovins, caprins et ovins de Guyane. Les sérums ont été collectés par la Direction de l'environnement, de l'agriculture, de l'alimentation et de la forêt entre 2015 et 2017 sur le littoral Guyanais, zone principale d’élevage de ruminants. L'analyse sérologique par ELISA indirect ciblait les IgG dirigées contre les antigènes de phase I et phase II de *C. burnetii.* Les résultats ont permis de calculer la prévalence apparente à l’échelle individuelle. À l’échelle des élevages, une méthode d'indexage originale a permis de classer d'abord les résultats individuels après transformation des taux d'anticorps détectés en variables qualitatives puis les proportions de ces variables par élevage. La mise en évidence d'un seul individu positif par élevage signifiait un statut positif d'exposition à la fièvre Q. Un score obtenu pour chaque élevage permettait d'apprécier son niveau d'exposition.

## Principaux résultats

L'analyse des données a révélé une prévalence apparente de 6,35 % (IC 95 % : 5,05-7,86%) pour toutes les espèces sur la période allant de 2015 à 2017, correspondant à un taux d'exposition inter-élevage de 37,61 % (32 élevages avec un score positif et 9 avec un score douteux). La prévalence apparente la plus élevée a été détectée en 2016 chez les bovins avec une prévalence des anticorps de 10,14 % (IC 95 % : 6,94-14,52%) et un taux d'exposition de 61,53 % au niveau inter-élevage (13 élevages avec un score positif et 3 avec un score douteux). L'analyse à l'aide des scores a montré une large variété des niveaux d'exposition.

## Discussion & Conclusion

Cette étude montre pour la première fois, et sur un échantillonnage d'envergure, l'exposition des animaux de rente en Guyane à *C. burnetii.* De plus cette étude a été réalisée à l'aide d'une technique mise en place selon les recommandations du Laboratoire national de référence pour la fièvre Q de l'ANSES. La circulation de cette maladie chez les bovins, caprins et ovins apparaît significative, à l'inverse des études disparates antérieures, et montre une variation temporelle et géographique. Le taux de prévalence indique que le rôle des élevages comme réservoir animal de *C. burnetii* est à prendre en compte pour les mesures de contrôle de cette zoonose dans le département. Cette étude épidémiologique transversale devrait être régulièrement répétée pour en apprécier les évolutions spatio-temporelles. Néanmoins, les investigations devront aussi porter sur les recherches d'autres réservoirs et sources de contamination possibles, et sur le décryptage des facteurs de risque de transmission, afin de mieux connaître l’épidémiologie de la fièvre Q en Guyane.

# Research article: Construction et évaluation d'un outil de contextualisation pour l'enseignement de la prise en charge des envenimations ophidiennes, dans le cadre de l'AFGSU

**Published:** 2022-06-30

**Authors:** Aurélia STANISLAS, Françoise ROUFOSSE, Myrna VICTOR, Anne-Lise MAITROT, Pierre CHESNEAU

**Affiliations:** 1 CESU 973, Centre d'enseignement des soins d'urgence de Guyane, Centre hospitalier de Cayenne, Guyane, France

## Contexte & Introduction

La prise en charge des plaies est prévue dans l'AFGSU (Attestation de formation aux gestes et soins d'urgence). En Guyane, les morsures de serpent sont envisagées dans ce module. Bien que son enseignement ait été compliqué par la médiatisation d'un décès en 2017, il a été actualisé.

## Méthodologie

Un questionnaire a été adressé aux formateurs pour avoir des précisions sur leur pratique afin d'envisager des propositions d'outils pédagogiques. Une évaluation de cet outil a été faite à distance.

## Résultats & Discussion

Deux techniques sont plus fréquemment utilisées : une discussion dirigée à partir de l'expérience de l'un des participants (7/19), ou une simulation (6/19). Le temps consacré à cet enseignement déborde le temps dévolu pour ce thème. La majorité des formateurs souhaitent disposer d'un outil pour l'aborder. Les formations CESU sont basées sur des pédagogies actives de l'adulte. Une vidéo est créée pour la contextualisation du module. En 3 minutes, on voit une famille en balade en forêt dont un jeune se fait mordre par un serpent. Le film s'achève sur le visage inquiet de la mère, avec la phrase « Qu'est-ce que je peux faire ? » Le formateur reprend cette question, ce qui permet de faire surgir les connaissances des participants. Après utilisation de la vidéo, 24 formateurs ont répondu au questionnaire. 70,8 % des formateurs considèrent la situation jouée comme tout à fait crédible et 25 % comme assez crédible. 62,5 % se servent systématiquement de cet outil et 20,8 % s'en servent souvent. Pour 25 % la discussion débute spontanément après la vidéo, et pour 75 % assez facilement. Pour 70,8 % les apprenants s'appuient sur les éléments du film pour la discussion. 95,9 % des formateurs considèrent que la vidéo facilite l'exploration des connaissances antérieures. 66,7 % trouvent particulièrement utile d'avoir un outil sous forme de film et 33,3 % trouvent cela assez utile.

## Conclusion

La morsure de serpent est un traumatisme cutané redouté en Guyane. Il est donc envisagé dans le cadre de l'AFGSU. La recherche des connaissances antérieures est souvent parasitée par l’émotion liée à la peur ancestrale du serpent. La réalisation d'un film a permis de faciliter l'expression du discours des participants quant aux actions qu'ils voudraient accomplir. Il reste maintenant à évaluer l'optimisation des apprentissages et le transfert des connaissances lors de situations réelles.

# Research article: Dermatologie médicale au Service d'accueil des urgences pédiatriques du Centre hospitalier de Cayenne, Guyane, France

**Published:** 2022-06-30

**Authors:** Victor TERTRE, Karim BENAMEUR, Jihen BAHRI, Alexis FREMERY

**Affiliations:** 1 Département universitaire de médecine générale, Université des Antilles, Pointe-à-Pitre, Guadeloupe, France; 2 Service des urgences pédiatriques, Centre hospitalier de Cayenne, Guyane, France; 3 Service des urgences, Centre hospitalier de Cayenne, Guyane, France

## Contexte

La Guyane est un territoire particulier de par sa localisation sud-américaine et son climat tropical. Sa population est caractérisée par une importante diversité culturelle et des inégalités socio-économiques marquées, notamment dans le domaine de l’éducation et de la santé. Dans les services d'urgences pédiatriques de France, les pathologies dermatologiques représentent une part importante des consultations avec une prédominance des dermatoses infectieuses et allergiques. L'objectif de ce travail est la description de l’épidémiologie des pathologies dermatologiques aux urgences pédiatriques du Centre hospitalier de Cayenne, Guyane, France (CHC).

## Méthodologie

Nous avons réalisé une étude rétrospective, descriptive, du 1er décembre 2020 au 31 mai 2021. Tous les patients ayant eu un diagnostic appartenant à la spécialité dermatologique ont été inclus. Nous avons exclu les patients dont l'orientation relevait d'une prise en charge chirurgicale au décours de la prise en charge aux urgences. Ce travail a été effectué conformément à la méthodologie de référence MR-004 de la CNIL, enregistré sous le numéro n° 2224516.

## Résultats

Au total, 334 dossiers de patients ont été analysés. La moyenne d’âge était de 3,4 ans, avec une discrète prévalence masculine (sexe ratio = 1,3). La majorité des patients inclus ne présentaient pas de réels critères de gravité (CIMU 4 = 93,1%). On retrouvait une consultation antérieure pour 16,2 % des patients avec une proportion adressée par un médecin représentant 6,3 %. L’étiologie infectieuse était la plus fréquente (57%), avec une importante prédominance de la gale représentant 19,3 % des cas, suivie de l'atopie (25,1%). La réalisation d'examens complémentaires concernait 6,6 % des patients et 3,9 % bénéficiaient d'une hospitalisation.

## Discussion & Conclusion

À l'instar de la France hexagonale, les dermatoses infectieuses représentent le premier motif de consultation dans le domaine dermatologique aux urgences pédiatriques, suivies par les dermatoses atopiques. On observe cependant une variation des agents infectieux responsables avec notamment une inversion des proportions virales et bactériennes, qui tend davantage vers celle de pays voisins comme l'Argentine. Ces consultations, souvent sans gravité, sont le reflet d'un manque d'accès aux soins, et d'une précarité économique et éducative marquée.

# Research article: Risques de réémergence de la fièvre jaune sur le plateau des Guyanes : une série de cas 1990-2020 et une revue de la littérature

**Published:** 2022-06-30

**Authors:** Caroline THOMAS, Céline MICHAUD, Mélanie GAILLET, Benoît de THOISY, Marcus LACERDA, Jean-Bernard DUCHEMIN, Sheila RODOVALHO, Stephen VREDEN, Ruth RAMOS, Mathieu NACHER, Cyril ROUSSEAU, Alice SANNA, Jacobus de WAARD, Fhabián Stevens CARRIÓNNESSI, Laurène TARDIEU, Maud LEKIEFFRE, Yann COSSARD, Félix DJOSSOU, Dominique ROUSSET, Hatem KALLEL, Loïc EPELBOIN

**Affiliations:** 1 Laboratoire hospitalo-universitaire de parasitologie-mycologie, Centre hospitalier de Cayenne, Guyane, France; 2 Pôle des Centres délocalisés de prévention et de soins, Centre hospitalier de Cayenne, Guyane, France; 3 Laboratoire des interactions virus-hôtes, Institut Pasteur de la Guyane, Cayenne; 4 Fundação de Medicina Tropical Doutor Heitor Vieira Dourado, International Center for Clinical Research in Malaria; 5 Responsable de l'Unité d'entomologie médicale, Institut Pasteur de la Guyane; 6 Pan American Health Organization, Unidade Técnica de Doenças Transmissíveis e Análise de Situação em Saúde; 7 Département de médecine interne, Academic Hospital Paramaribo, Suriname; 8 Directrice du National Care and Treatment Center, Guyane; 9 Centre d'investigation clinique, CIC INSERM 1424, Centre hospitalier de Cayenne, Guyane, France; 10 Santé publique France, antenne Guyane; 11 Departamento de Tuberculosis, Instituto de Biomedicina, Universidad Central de Venezuela, Caracas, Venezuela; 12 Health Sciences School, University of Oriente, Bolívar Nucleus / Biomedical Research and Therapeutic Vaccines Institute, Ciudad Bolívar, Venezuela; 13 Service de réanimation néonatale, Centre hospitalier de Cayenne, Guyane, France; 14 Service des maladies infectieuses et tropicales, Centre hospitalier de Cayenne, Guyane, France; 15 Service de réanimation, Centre hospitalier de Cayenne, Guyane, France

## Contexte & Introduction

Le virus de la fièvre jaune (FJ) cause des épidémies mortelles en Amérique du Sud. La vaccination a permis une quasi-éradication sur le bouclier guyanais (BG), mais quelques cas font craindre une réémergence. L'objectif de cette étude est de recueillir tous les cas de FJ identifiés sur le BG, une région du nord-est de l'Amérique du Sud comprenant l’État de Bolívar (Venezuela), le Guyana, le Suriname, la Guyane française et l'Amapá

(Brésil).

## Méthodologie

Inclusion des personnes vivant ou voyageant sur le BG avec des symptômes compatibles avec la FJ confirmés par PCR entre 1990 et 2020. Les dossiers médicaux des cas diagnostiqués à l'Institut Pasteur de Guyane ont été étudiés, les infectiologues des pays avoisinants interrogés et la littérature médicale analysée.

## Principaux résultats

8 patients présentant une FJ ont été identifiés : 5 en Guyane, 1 cas américain, 1 cas chez une Hollandaise au retour du Suriname et 1 cas au Venezuela. Seuls le patient vénézuélien et la patiente hollandaise ont survécu. L’âge médian est de 45 ans; 2 d'entre eux étaient des Français amérindiens du Maroni, 2 étaient des orpailleurs brésiliens illégaux en Guyane, 1 était un touriste suisse vivant en Guyane, et le dernier était un touriste américain en vacances au Venezuela. Le tableau clinique était celui d'une hépatite fulminante évoluant vers une défaillance multiviscérale. Le décès dans 6/8 patients survenait dans un délai médian de 7,5 jours. 4/8 avaient des co-infections – le jeune Amérindien avait le SARS-CoV-2. 2/8 étaient vaccinés : le jeune Amérindien avait reçu une dose du vaccin contre la FJ dans l'enfance. L'autre Français avait été vacciné 13 ans plus tôt.

## Discussion & Conclusion

La FJ est rare mais sa circulation persiste sur le BG. Le taux de mortalité moyen (6/8 = 75%) est élevé par rapport aux études brésiliennes (32,5%). 2/8 (25%) des patients avaient reçu le vaccin : quelle est la pertinence de la dose unique ?

La gravité mise en évidence est probablement liée à un premier contact médical trop tardif et/ou à un retard diagnostic important. Enfin une couverture vaccinale suffisante doit être maintenue, pour les populations locales comme pour les sujets voyageant sur le BG.

# Research article: Spectre des pathologies dermatologiques dans les communes Bushinengués du Maroni « BUBASKIN »

**Published:** 2022-06-30

**Authors:** Julie VALENTIN, Florence NIEMETZKY, Mélanie GAILLET, Céline MICHAUD, Aurel CARBUNAR, Magalie DEMAR, Pierre COUPPIÉ, Romain BLAIZOT

**Affiliations:** 1 Service de dermatologie, Centre hospitalier de Cayenne, Guyane, France; 2 Centres délocalisés de prévention et de soins (CDPS), Centre hospitalier de Cayenne, Guyane, France; 3 Tropical Biome and Immunophysiopathology (TBIP), Université de Lille, CNRS, INSERM, Institut Pasteur de Lille, U1019-UMR9017-CIIL Centre d'infection et d'immunité de Lille, Centre hospitalier de Cayenne,, Université de Guyane, France; 4 Laboratoire de parasitologie, Centre hospitalier de Cayenne, Guyane, France

## Contexte & Introduction

Eu égard à leurs caractéristiques génétiques, leur mode de vie traditionnel dans les forêts équatoriales et leur exposition importante à des maladies infectieuses, les Bushinengués sont susceptibles de développer un spectre particulier de dermatoses. L’épidémiologie des pathologies dermatologiques des Bushinengués n'est pas décrite.

## Méthodologie

Cette étude rétrospective concernait tous les patients ayant consulté dans les Centres délocalisés de prévention et de soins (CDPS) de Papaïchton, Maripasoula, Apatou et Grand Santi entre le 5 octobre 2017 et le 30 juin 2020. Étaient inclus les patients ayant consulté pour un motif dermatologique selon le CIM-10. Les informations des dossiers médicaux étaient vérifiées par un dermatologue afin de corriger les erreurs de cotation.

## Principaux résultats

Un total de 4 741 patients formait la population de l’étude, pour 6 058 diagnostics dermatologiques différents qui ont motivé 7 253 consultations. Les diagnostics les plus fréquents étaient les infections cutanées (71,6%), devant les dermatoses inflammatoires (9,8%) et les morsures et envenimations (4,6%). Les 3 diagnostics les plus fréquents étaient la gale (902 cas, 14,6%), les abcès et les impétigos. Les maladies tropicales négligées (MTN) étaient prévalentes, puisque nous rapportons 13 cas de lèpre et 63 leishmanioses. Avec une prévalence proche de celle de la population générale française, la dermatite atopique était la dermatose inflammatoire la plus fréquente (2,5%). A contrario, nous notions peu de tumeurs cutanées, et 3 cas d'albinisme.

## Discussion & Conclusion

Cette étude a permis de mettre en évidence le poids des maladies infectieuses chez les Bushinengués du Maroni. Toujours prévalentes, les MTN telles que la gale, la lèpre, la leishmaniose cutanée devraient être la cible d'actions de santé publique. Deux cas de mélanome acro-lentigineux à des stades avancés sont rapportés, montrant la faible fréquence et le manque de sensibilisation à cette tumeur des patients et de leurs soignants. La prise en compte de ces résultats devrait permettre d'optimiser la prise en charge des médecins dans les CDPS.

# Research article: Évaluation de la douleur chez le patient drépanocytaire : étude exploratoire auprès des soignants du Centre intégré de drépanocytose de Guyane en 2021

**Published:** 2022-06-30

**Authors:** Yabo ZITTI, Nathalie DEVILLIERS

**Affiliations:** 1 Centre intégré de drépanocytose de Guyane, Centre hospitalier de Cayenne, Guyane, France; 2 Institut de formation en soins infirmiers de Cayenne (I.F.S.I.), Université de Guyane

## Contexte & Introduction

La drépanocytose est la maladie génétique la plus répandue et touche 2,3 % de la population mondiale. Les départements d'outre-mer et l’Île-de-France sont les plus touchés en France. En Guyane, 10 % de la population portent le gène et 0,5 % sont atteints de la maladie. Bien que la drépanocytose se manifeste par l'anémie, des infections et des complications parfois graves, la prégnance de la douleur dans la crise résume souvent à elle seule la maladie. L'objectif de ce travail de fin d’étude est de savoir si l’évaluation de la dou-leur révèle ce que le patient drépanocytaire ressent et exprime.

## Méthodologie

Nous avons réalisé une enquête de terrain basée sur des entretiens semi-directifs auprès de trois infirmières et un médecin, tous travaillant au Centre intégré de drépanocytose de Guyane.

## Résultats

50 % des soignants estiment que l’évaluation de la douleur ne révèle pas toujours ce que le patient ressent et exprime. Entre le ressenti et l'expression, plusieurs facteurs peuvent interférer tels que le seuil de sensibilité, l'angoisse, l'anxiété, le vécu, la culture…

Deux infirmières sur trois ont expliqué que le vécu ou la culture du soignant peut avoir une in-fluence dans l’évaluation de la douleur.

Cette enquête exploratoire a permis d’élaborer un protocole de recherche en se basant sur la question suivante : En quoi la prise en compte conjointe de l'expression et du ressenti du patient peut améliorer l’évaluation de sa souffrance ?

## Discussion & Conclusion

Ces entretiens autour de l’évaluation de la douleur confrontés à la revue de littérature démontrent qu'en plus des compétences infirmières, cette évaluation nécessite de l'empathie, une neutralité bienveillante et une écoute active. L'utilisation d'une échelle descriptive et spécifique à la drépanocytose pourra permettre au patient de décrire sa douleur. La communication dans une relation d'aide permettra au patient d'exprimer son ressenti et sa souffrance pour une prise en charge efficace de cette douleur.
